# The interaction of CpEBF1 with CpMADSs is involved in cell wall degradation during papaya fruit ripening

**DOI:** 10.1038/s41438-018-0095-1

**Published:** 2019-01-01

**Authors:** Xiaochun Ding, Xiaoyang Zhu, Lanlan Ye, Shuangling Xiao, Zhenxian Wu, Weixin Chen, Xueping Li

**Affiliations:** State Key Laboratory for Conservation and Utilization of Subtropical Agro-Bioresources/Guangdong Provincial Key Laboratory of Postharvest Science of Fruits and Vegetables, College of Horticulture, South China Agricultural University, Guangzhou, 510642 China

**Keywords:** Transcriptional regulatory elements, Plant signalling

## Abstract

Ethylene plays a pivotal role in climacteric fruit ripening; whereas 1-MCP, a non-toxic antagonist of ethylene, prevents ethylene-dependent responses and fruit ripening. In this study, a short-term treatment (1 h) with 400 nL L^−1^ 1-MCP delayed the ripening of harvested papaya. However, long-term application of 1-MCP (400 nL L^−1^, 16 h) resulted in abnormal fruit ripening, with the fruits exhibiting normal yellowing without softening, significantly higher cellulose and lignin contents, and intact cell walls (CW). Furthermore, we found that long-term treatment with 1-MCP significantly inhibited the expression of *CpEBF1*, an EIN3-binding F-box-1 gene. A protein interaction analysis using yeast two-hybrid, BiFC and GST pull-down assays showed that CpEBF1 interacts with the CpMADS1/3 and CpEIL1 proteins. The interaction of CpEBF1 with CpMADS1/3 further activated the activities of CW-degradation gene promoters. Subcellular localization showed that these proteins were localized in the nucleus. Additionally, the expression levels of *CpMADS1/3*, *CpEIL1*, and several CW-degradation-related genes were significantly downregulated by long-term 1-MCP treatment. Therefore, we propose that the inhibited expression of *CpEBF1* and *CpMADS1/3* resulted in the repressed activation of CW-degradation-related genes via their interaction, thereby resulting in fruit softening disorders.

## Papaya: Anti-ripening treatment prevents degradation of cell wall

Long-term treatment with an anti-ripening agent inhibits the expression of regulatory genes that normally break down the cell wall of the papaya fruit. A team from Guangzhou’s South China Agricultural University led by Xueping Li and Weixin Chen applied a hormone inhibitor to papayas at the breaker stage of fruit ripening. 1 h of treatment delayed ripening, whereas 16 h of treatment caused the fruit to become rubbery, with significantly higher levels of cellulose and lignin, both structural components of the cell wall. The researchers identified two genes with reduced expression following extended hormone-blocking treatment. Both normally encode proteins that aid in degrading the cell wall to promote fruit ripening. The findings thus offer a molecular explanation for why misuse of anti-ripening agents on papaya fruits can lead to undesirable characteristics.

## Introduction

The papaya is a major fruit in tropical and subtropical regions, and it is known for its rich nutritional value^1^. However, the release of ethylene immediately peaks during papaya fruit ripening, resulting in rapid softening and a deterioration of the fruit quality, which in turn dramatically shortens its storage period and thus limits its handling and transportation^2^. Because papaya is a typical climacteric fruit, ethylene plays a vital role in the ripening of its fruit^3^. Postharvest technologies for controlling ethylene and the ethylene pathway have been studied extensively^4,5^. As an ethylene receptor inhibitor, 1-methylcyclopropene (1-MCP) has been employed to increase the shelf-life of various climacteric and non-climacteric fruits ^6^. However, the practical application of 1-MCP continues to be associated with various challenges. For example, 1-MCP may trigger the production of undesirable volatiles and cause abnormal fruit coloration and softening in apples^7^. Unsuitable 1-MCP (500 nL L^−1^, 16 h) treatment may cause bananas to stay green or ripen with uneven color^8,9^. In addition, 1-MCP has also been applied to maintain the papaya fruit quality and extend its shelf-life^10^. However, unsuitable 1-MCP treatment (long-term or high concentrations) tends to cause an elastic state or “rubbery” texture in papaya, and its underlying mechanism remains unclear.

The effects of 1-MCP are thought to involve ethylene receptors that prevent ethylene from binding to its receptors and influence fruit ripening^11^. Ethylene signal transduction has been extensively studied in the model plant *Arabidopsis*. Changes in ethylene receptors stimulate the expression of various genes involved in the ethylene signaling pathway and downstream, thereby regulating fruit ripening^12^. EIN3 binding F-box-1/2 (EBF1/2) plays important and similar roles in the ethylene signaling transduction pathway in *Arabidopsis*^4^; namely, they target EIN3, the master transcriptional regulator of the ethylene response, for proteasomal degradation^13^. The function and characterization of the *EBF* genes have been studied in only a few fruit species, such as tomatoes^14^, bananas^15^, and apples^16^. Silencing the *SlEBF1*/*2* gene causes a constitutive ethylene reaction phenotype, thereby accelerating tomato ripening^14^. The *MaEBF1* and *MaEBF2* genes showed different expression patterns in bananas, in which *MaEBF1* is constitutively expressed and its transcript abundance changes slightly with ethylene and 1-MCP treatment, whereas *MaEBF2* expression is significantly upregulated by ethylene and inhibited by 1-MCP treatment^15^. In apples, *EBF1* and *EBF2* are significantly upregulated at the fruit ripening stage, and *EBF1* negatively regulated *PG1* by interacting with EILs^16^. The operations of EBF in terms of ethylene response have already been examined in *Arabidopsis* and tomato, but the current understanding of its role in the ripening of other economically important fruit, such as papaya, is limited.

The *MADS-box* gene family-encoded proteins are important transcription factors in plants, and they are evolutionarily conserved^17^. Numerous studies have showed that MADS proteins have participated in various evolutionary, developmental and metabolic processes in plants^18,19^. An increasing number of studies have recently focused on the roles of the MADS-box transcription factor in fruit ripening. In tomatoes, SlMADS-RIN is a classical MADS-box protein that positively regulates fruit ripening^20^. Ripening inhibitor (RIN) has been reported to regulate various ripening processes directly, such as the ethylene response, CW metabolism, aromas and pigments, and energy metabolism^18^. Reduced *MaMADS3* expression was observed in *MaMADS1* and *MaMADS2*-repressed lines due to the reduction in ethylene production^19^. Previous studies have examined the relationship between MADS and CW-modification genes in tomatoes. *MADS-RIN* mutants inhibit the expression of the *EXP1* gene, resulting in a failure to soften^20^. The silencing of the *FUL1* and *FUL2* (*FRUITFULL-like MADS-box*) genes results in the suppression of CW modifications, thereby inducing abnormal fruit ripening^21^. MADS-box genes acting upstream of the ethylene pathway primarily regulate the ethylene synthesis that controls fruit ripening in turn^22,23^. However, the direct relationship between MADS and the ethylene signal transduction pathway elements has not been investigated to date, and the interaction between CpMADS1/3 and CpEBF1 remains unclear.

From a microscopic point of view, softening is due to the dissolution of the CW middle layer. Fruit softening causes the primary CW to become loose until it degrades, resulting in a decrease in cell–cell interactions and hardness^24^. Different enzymes are involved in CW degradation, such as pectin methylesterase (PME), polygalacturonase (PG), cellulase (CX), and pectate lyase (PL), and other enzymes, which all act in an interdependent manner^25^. Recent work reported that *PG*s and *PME*s showed various expression profiles among three soft-fleshed and rapidly softening pear cultivars, thereby confirming their important roles in fruit ripening^26^. Several CW modifying genes were identified in papaya, such as *PG*s and *PME*s^27^, but their expression regulation in relation to papaya fruit ripening is still not clear. In a previous work, the papaya *CpERF9*, a transcriptional repressor of the cell wall modification gene, was found to control fruit ripening and softening by directly binding to the promoters of *CpPG5* and *CpPME1/2*^28^. Although several softening genes have been isolated, the transcriptional regulation of these softening enzymes and genes in 1-MCP-induced papaya softening disorders remains unclear. Therefore, we hypothesize that unsuitable long-term 1-MCP treatment results in softening disorders that are associated with the transcriptional regulation of CW genes. This work will provide a better understanding of papaya fruit ripening at the physiological and molecular levels, which will help to extend its shelf-life and maintain quality.

## Materials and methods

### Plant materials and treatments

Papaya fruits (*Carica papaya* L., cv. ‘Suiyou-2′) at the color break stage (5% < peer color < 15% yellow)^29^ were obtained from a local commercial farm in Guangzhou, South China. Fruits with similar sizes that were free of blemishes were selected and cleaned with water, immersed in 0.2% (w/v) hypochloride solution for 10 min, and then dipped in 500 mg mL^−1^ mixture solutions of iprodione and prochloraz. After the samples air-dried at 22 °C, three different treatments were performed on them, namely, 400 nL L^−1^ of 1-MCP (Kuaida, Jiangsu, China) for 1 h and 16 h and a control treatment. For the 1-MCP treatments, the fruits were fumigated with 400 nL L^−1^ 1-MCP for 1 h or 16 h in a closed foam box, and then they were treated with 1000 μL L^−1^ ethephon for 1 min and ripened at 22 °C. For the control treatment, the fruits were sealed in a foam box for 16 h without 1-MCP, then treated with 1000 μL L^−1^ ethephon for 1 min and ripened at 22 °C. Pulp samples were collected from the middle part of each fruit, frozen in liquid nitrogen, and then stored at −80 °C. For the control treatment, samples were collected at 0, 1, 2, 4, and 6 days. For the 1-MCP treatments at 1 h and 16 h, samples were taken at 0, 1, 2, 4, 6, 8, 11, and 14 days after treatment. All the treatments were conducted using three biological replicates, each of which consisted of 150 fruits.

### Fruit firmness, coloring index assessment, respiration, and ethylene production

The pulp hardness was measured using a Harness Tester 5542 (Instron, Norwood, NT, USA) equipped with a columniform planar plunger (8 mm in diameter). A small piece of papaya peel was torn off, and the hardness at nine different points in the middle of the fruits was determined^29^. The fruit coloring index was evaluated over a range from 1 to 6 as follows: 1, entirely green; 2, 0 < yellow < 25%; 3, 25% < yellow < 50%; 4, 50% < yellow < 75%; 5, 75% < yellow < 100%; and 6, 100% yellow. Coloring index = ∑(Coloring grade × Number of fruits)/Total number of fruits^29^. The respiration rate and ethylene production were determined according to Fu et al.^30^. Five fruits per treatment were weighed and placed individually in an airtight container equipped with a rubber stopper for 2 h at 22 °C. A triplicate sample of 1 mL of headspace gas was used for the ethylene and respiration rate determinations.

### Ultrastructure and microstructure observations of the fruit CW using a transmission electron microscopy (TEM) and safranin staining

Pulp tissues (1 mm^3^) close to the peel and from the middle part of the fruit from different storage periods were collected and fixed with 2.5% glutaraldehyde and 1% osmium tetroxide, then treated as follows: washed with PBS buffer, subjected to an ethanol gradient elution, embedded in SPI812 resin, ultrasonically sliced with Ultracut Uct (Leica, Solms, Germany), and then stained with uranyl acetate and lead citrate. The images were captured using a TEM (TECNAI-12, PHILIPS, Amsterdam, Holland) using 2 KV of accelerating voltage^31^.

The microstructure of the fruit cells was assessed as previously described^31^. First, 1 mm^3^ of pulp tissues around the middle part of the fruit and close to the peel was fixed in the solution (40% formaldehyde, 70% ethanol, and glacial acetic acid) for 48 h. The fruit tissues were then dehydrated across an ethanol gradient. The samples were embedded in 1% paraffin and solid green (FCF) (2.5 mg/L solid green in 60% ethanol) and then rinsed with 100% ethanol. After being dried, the samples were imaged using a U-TV 0.63XC instrument (Olympus, Tokyo, Japan).

### Measuring the activity of the CW-degradation-related enzymes PME, PL, PG, and CX

The activity determination of the CW-degradation-related enzymes PME, PL, PG, and CX was performed according to Guo et al.^32^.

### Total RNA isolation and reverse transcription cDNA synthesis

The total fruit RNA was extracted using the hot borate method^33^. A quantity of 1–2 μg of RNA was reverse-transcribed using the ReverTra Ace RT-qPCR kit (Bio-Rad, USA).

### Gene expression analysis

Gene expression analyses were conducted by quantitative real-time PCR (RT-qPCR) analysis. All the RT-qPCRs were conducted following the optimized methods in our previous study^34^. The primers used in this study are listed in Supplementary Table 1. *CpTBP1* was selected as a reference gene in papaya as previously validated. The relative expression was calculated using the 2^–ΔΔCt^ method ^35^. Three biological replicates were used for the analysis.

### Construction of a cDNA library

Mixed RNA samples of fruits were treated with 1-MCP for 1, 2, 4, and 8 days for the construction of a cDNA library. The RNA was subjected to DNase I digestion using an RNase-free kit (Promega, Madison, WI, USA). The total mRNA was isolated according to the manufacturer’s protocol (Promega, USA). The SMART™ cDNA Library Construction kit was used to construct the cDNA library (Clontech, Fitchburg, WI, USA). A Yeast Maker Transformation System 2 kit (Clontech, USA) was used for yeast transformation. Yeast two-hybrid (Y2H) screening (Clontech, USA) was performed using standard procedures, followed by the screening of the cDNA library. The cDNA library contains more than 2 million independent clones, of which more than 1 million independent clones were screened. Yeast plasmid isolation was performed using an Easy Yeast Plasmid Isolation Kit (Clontech, USA) with minor modifications.

### Y2H screening and transcriptional activation

The Y2H experiment was conducted using the Matchmaker Gold Yeast Two-Hybrid System (Clontech, USA). The ethylene signal transduction genes *CpEBF1/2*, *CpEIL1/2/3/4*, and *EIN3a/b* were each ligated to a pGBKT7 vector. The yeast cells were grown on a synthetic medium without tryptophan (Trp), histidine (His), and adenine (Ade) and containing 125 μM aureobasidin A. Colonies were grown on the synthetic medium and stained with the chromogenic substrate X-α-gal, which turned blue with transactivation activity.

Toxicity testing indicated that the full-length CpEBF1 protein does not cause toxic effects in the yeast host cell (data not shown). Then, the CpEBF1 protein was used as the bait protein in the cDNA library screening. CpEBF1 was ligated to the pGBKT7 vector and then used as a bait to capture the interacting proteins in the cDNA library. CpMADS1/3 and CpEIL1 were the prey proteins. Full-length *CpMADS1/3* and *CpEIL1* were each cloned into a pGADT7 vector. To identify the CpMADS1/3 domain that interacts with CpEBF1, four different domains were identified and cloned into the pGADT7 vector. The yeast strains were co-transformed with CpEBF1 + CpMADS1/3 or CpEBF1 + CpEIL1. PGBKT7-lam + pGADT7-T or pGBKT7-53 + pGADT7-T was used as a negative or positive control. All the primers used in the Y2H assay are presented in Supplementary Table 2.

### Bimolecular fluorescence complementation assays (BiFC)

The full-length cDNAs of *CpEBF1*, *CpMADS1/3*, and *CpEIL1* were fused with binary pBIFC vectors using Gateway technology, and they contained the amino-terminal fragment of the EYFP fluorescent protein or the carboxy-terminal fragment of the ECFP fluorescent protein (YFP^N^ and ECFP^C^). ECFP^C^ is modified from YFP^C^ to enhance the fluorescent signals in the BiFC assay. All the constructs were transformed in *Agrobacterium* strain GV3101. Different combinations of these constructs were mixed at a 1:1 OD_600_ ratio and injected into the epidermal cells of 3- to 4-week-old *Nicotiana benthamiana* plants. After 36–72 h, fluorescent signals were observed under a fluorescence microscope (Zeiss Axioskop 2 Plus, Leica, Solms, Germany), and the spectral detector was set to have excitation and emission wavelengths of 515 nm and 540 nm, respectively.

### GST pull-down assay to verify protein interactions

Recombinant GST-tagged CpEBF1, His-tagged CpMADS1/3, and CpEIL1 proteins were produced. The fusion construct was transformed into BM Rosetta (DE3) cells and then induced with 1.0 mM IPTG. CpEBF1-GST was incubated at 28 °C for 6 h, and CpEIL1-His was induced at 37 °C for 8 h. CpMADS1/3 was induced overnight at 16 °C, whereas no purified protein was obtained from CpMADS3. Approximately 5 µg of purified His-fusion protein was bound to Ni-NTA His-binding resin (Clontech, USA). CpEBF1-GST proteins were purified with a GST purification kit (Clontech, USA). GST fusion proteins containing protein GST- and His- antibodies were used for western blot analysis. All of the primers used in the GST pull-down assay are shown in Supplementary Table 3.

### CpMADS1/3 protein evolutionary and sequence analyses

A sequence alignment was performed using CLUSTALW 1.83, and a phylogenetic reconstruction was conducted using MEGA5 software. The GenBank accession numbers of the MADS for phylogenetic analysis are listed in Supplementary Table 4. The protein alignment diagram was drawn using DNAMAN software.

### Subcellular localization analysis

The full-length cDNAs of *CpEBF1*, *CpMADS1/3*, and *CpEIL1* were each cloned into a pENTR/D vector (Invitrogen, USA). The pENTR vectors were incubated with a pGWB5 vector together with an LR clonase enzyme (Invitrogen, USA) to generate the CpEBF1-GFP, CpMADS1/3-GFP, and CpEIL1-GFP fusion proteins. The leaves of 3- to 5-week-old *N. benthamiana* plants were infiltrated with each of the GV3101 strains containing the above pGWB5 constructs. The localization of fluorescent proteins was observed 36–72 h after treatment using a fluorescence microscope (Zeiss Axioskop 2 Plus, Leica, Solms, Germany). All of the transient expression experiments were repeated at least thrice. To obtain an empty pGWB5 vector that carries free GFP, the pGWB5 vector was incubated with a self-ligated pENTR/D vector and LR clonase, which generates an empty pGWB5 vector without the ccdb gene. The primers used in the subcellular localization analysis are listed in Supplementary Table 5.

### Transient expression assays

The total genomic DNA was extracted from young papaya leaves using the DNeasy Plant Mini Kit (Qiagen, USA) according to manufacturer’s instructions. The promoters of *CpPG1/2*, *CpPME1/2*, and *CpEXP1/2* were isolated from the papaya genome (ftp://ftp.jgi-psf.org/pub/compgen/phytozome/v9.0/Cpapaya) and cloned by PCR amplification (the primers are shown in Supplementary Table 6).

The promoters of *CpPME1/2*, *CpEXP1/2*, and *CpPG1/2* were cloned into a pGreenII 0800-LUC double-reporter vector, whereas those of *CpEBF1*, *CpMADS1/3*, and *CpEIL1* were cloned into the pGreenII 62-SK vector as effectors. The LUC activity was normalized using the REN activity. The double-reporter vector contained GAL4-LUC and REN, an internal control, driven by a 35S promoter. The constructed effector and reporter plasmids were co-transformed into tobacco using *A. tumefaciens* strain EHA105 with a pSoup vector. The LUC and REN luciferase activities were determined using the Dual Luciferase Assay Kit (Promega, Madison, WI, USA) and analyzed on a Luminoskan Ascent microplate luminometer (Thermo Scientific, USA) according to the manufacturer's instructions. The LUC/REN ratio was then calculated. At least six transient assay measurements were included for every experiment.

### Statistical analysis

Each treatment comprised three independent biological replicates. The collected data were then subjected to variance analyses^36^. Charts were drawn using SigmaPlot 12.0 software. Duncan’s multi-range test was used to compare the means among various groups. The data were expressed as the means ± standard deviation (SD). The least significant difference (LSD) at the 5% level was analyzed using SPSS 21.0 software^37^. Significant differences between groups were confirmed when *P*-value <0.05^38^.

## Results

### Physiological characterization of fruits during storage and fruit ripening

**Fig. 1:**
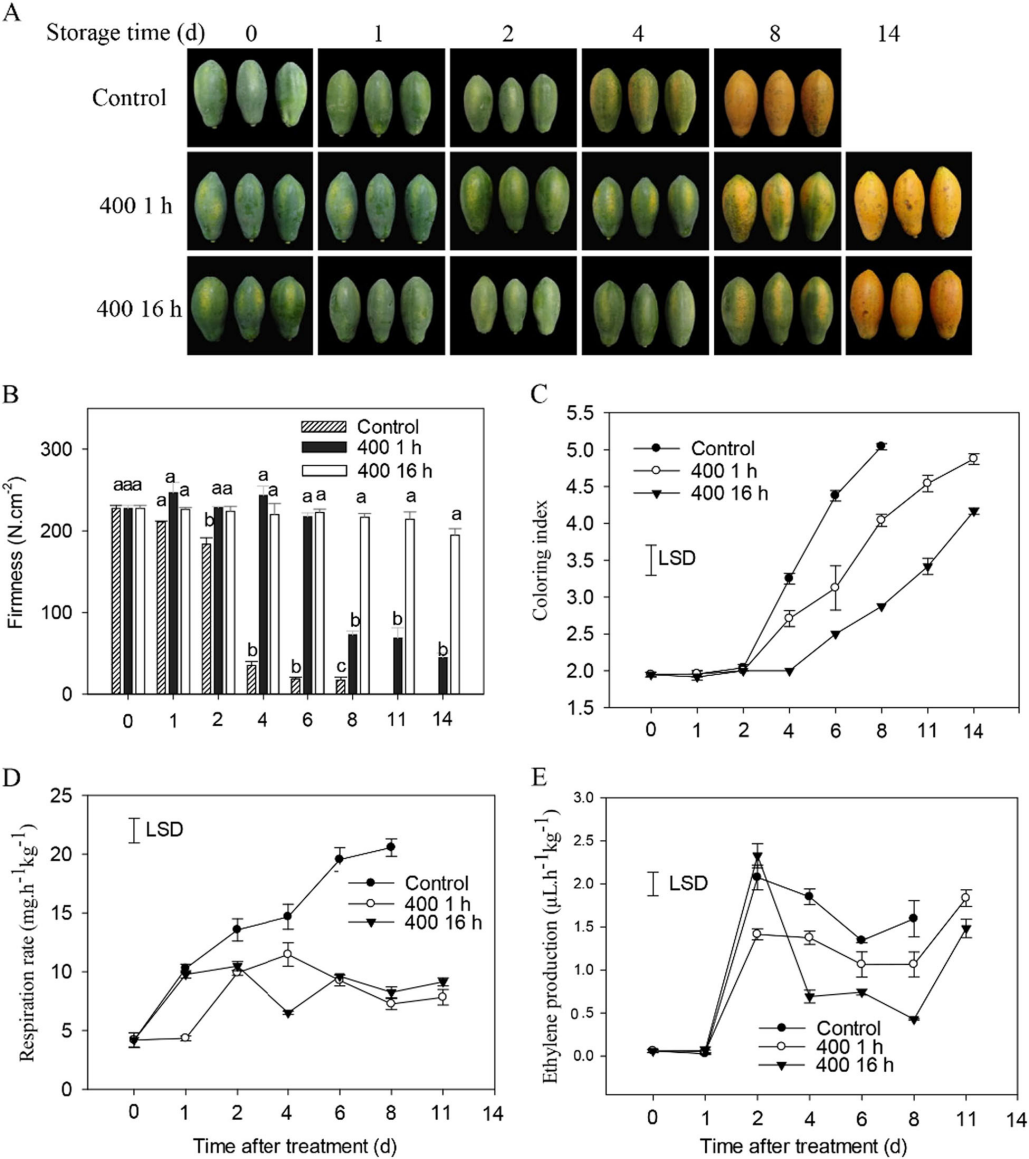
Effects of 1-MCP treatment on the firmness, coloring index, respiration rate, and ethylene production in papaya fruits. **a** 1-MCP-treated papaya fruits during storage. **b** Changes in fruit firmness. **c** Changes in coloring index. **d** and **e** Fruit respiration rate and ethylene production; for 400 16 h, the fruits were fumigated with 400 nL L^−1^of 1-MCP for 16 h followed by 1000 μL L^−1^ ethephon treatment. For 400 1 h, fruits were fumigated with 400 nL L^−1^ of 1-MCP for 1 h followed by 1000 μL L^−1^ ethephon treatment. The control fruits were sealed in a foam box for 16 h without 1-MCP and then treated with 1000 μL L^−1^ ethephon for 1 min. A total of three biological replicates were analyzed, and the vertical bars indicate the SE. Different letters above the bars represent a significant difference at the 5% level between treatments. The LSD at *P* = 0.05 was calculated to compare the differences between the means of various treatments

Our previous study showed that treating with high concentrations of 1-MCP (700 nL L^−1^, 16 h) resulted in the abnormal ripening of the papaya fruit (data not shown). Treatments involving 1-MCP at the same concentration (400 nL L^−1^) and for different durations (1 h or 16 h) were performed in this study. Both 1-MCP treatments delayed fruit ripening; however, unsuitable 1-MCP (400 nL L^−1^, 16 h) treatment resulted in a ‘rubbery’ texture, which is considered as a type of fruit ripening disorder. Figure 1 shows that the papaya fruits softened 4 days after treatment, and the coloring indices rapidly increased on the 4th day of treatment in the control group. However, the 1-MCP treatments delayed fruit ripening, including fruit coloring and softening (Fig. 1a–c), particularly for the 16 h 1-MCP treatment. The fruit firmness dropped sharply at the 8th day after treatment with 1-MCP for 1 h, whereas those subjected to 16 h of 1-MCP treatment remained firm during the entire storage period, even at the end of ripening (Fig. 1b). Both 1-MCP treatments significantly reduced the peak respiration rates (Fig. 1d) as well as the ethylene production during extended storage (Fig. 1e). The unsuitable 1-MCP treatment shows more severe repression for fruit respiration and ethylene production.

### Ultrastructural observations of the fruit CW and paraffin section observation of cellulose and lignin changes

**Fig. 2:**
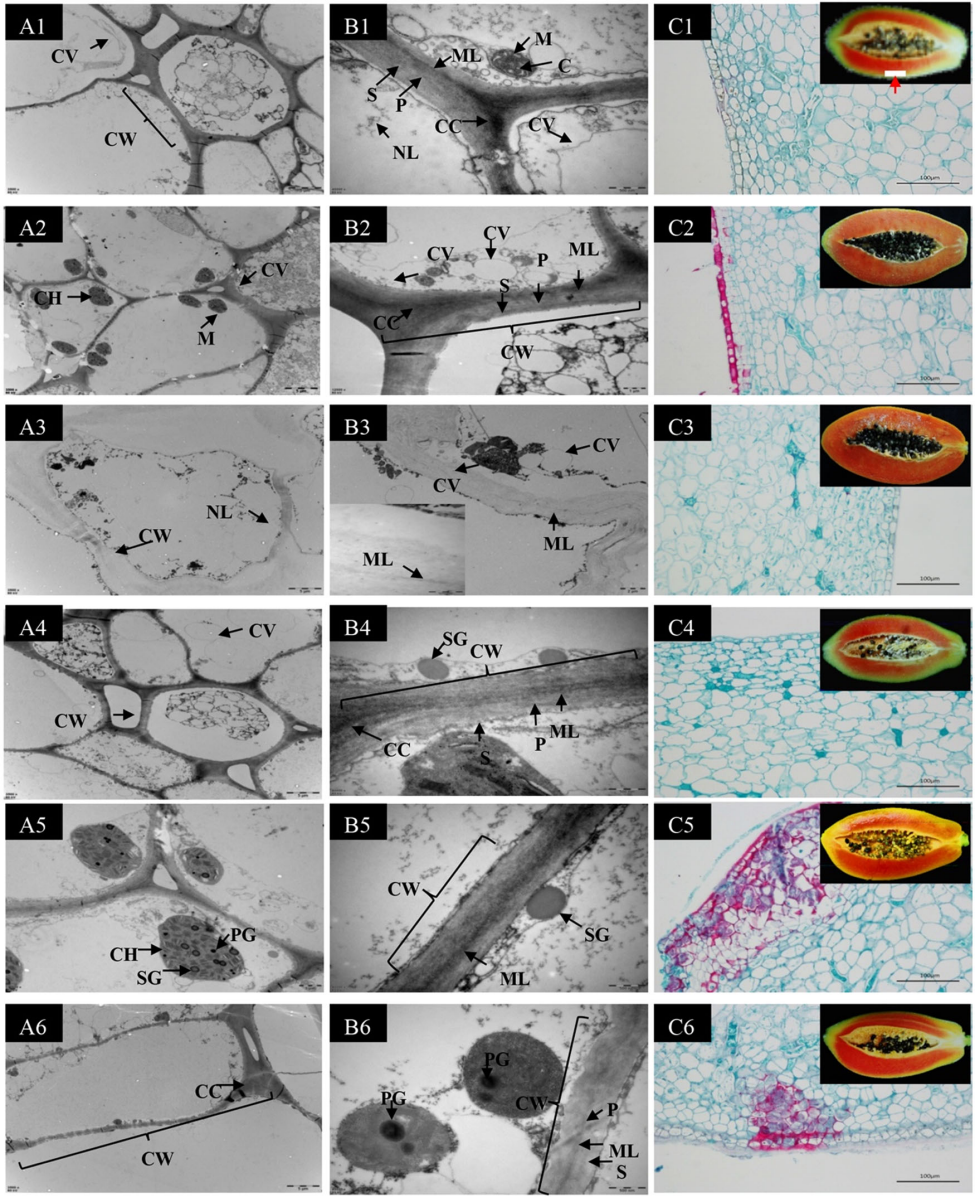
Ultrastructural and microstructural assessment of fruit cell walls by transmission electron microscopy (TEM) and paraffin sectioning. Day 0 after harvest (DAH) (A1, B1, and C1); Control group, 4 DAH (A2, B2, and C2); Control group, 8 DAH (A3, B3, and C3); 1-MCP for 16 h, 4 DAH (A4, B4, and C4); 1-MCP for 16 h, 8 DAH (A5, B5, and C5); 1-MCP for 16 h, 11 DAH (A6, B6, and C6); the A and B images were capture using TEM. **a** Bars = 5 μM; **b** Bars = 0.5 μM; **c** images were taken using paraffin section analysis with safranin and solid green. Safranin was used to stain the lignin, and solid green was used to stain the cellulose. Bars = 100 μM; CWcell wall; PM plasma membrane; ML intercellular layer; SW secondary wall; PW primary wall; SG starch granules; P primary wall; S secondary cell; MF microfiber; M mitochondrion; and CH chloroplast. The white rectangle in C1, around the middle part of the papaya pulp close to the peel, was studied under a microscope

The microscopic effects of 1-MCP treatment on the pulp tissues were assessed by TEM and paraffin sectioning. Figure 2 shows that unsuitable 1-MCP (400 nL L^−1^_,_ 16 h) prevents fruit softening by inhibiting the degradation of the microfilament in the middle layer of the CW and accelerating lignin accumulation. On the day of harvest, the fruits showed a complete CW structure, with alternating light and dark structures inside the CW and plasma membrane located next to the CW. Microfibrous filaments (MF) with uniform thickness that were arranged in an orderly, consistent manner were observed (Fig. 2a1, b1). This structure apparently maintains the strength and toughness of the cells, thereby resulting in firm flesh. The mitochondria (M) were intact, round, or oval in shape and were clearly visible in the medial fold, with the stroma visible within the mitochondria (Fig. 2b1). Cellulose was evenly distributed across the CW and trace amounts of lignin were observed (Fig. 2c1). After storage for 4 days, the CW structure of the control and 1-MCP-treated fruits remained essentially intact, the arrangement of fiber filaments still alternated between light and dark, and the intracellular organelles were clearly visible (Fig. 2a2, b2, and c2). However, compared to the 1-MCP treatment group, the MFs were slightly degraded and the number of mitochondria and pseudolipid particles increased, and the outer wall of the cell was partially dissolved. Lignin accumulated significantly on the outermost pulp cells in the control fruits (Fig. 2 a4, b4, and c4). A significant decrease in fruit firmness was observed in the controls on the eighth day of storage, the CW has completely degraded, and the middle layer of the pulp tissues had almost completely degenerated. The middle layer of the CW became transparent, and the fibers were fragmented. The edge of the CW was diffuse, and no intact organelles were observed. A disorganized, swollen cellular structure was detected, with an intense green hue indicating cellulose degradation. The cellulose and lignin had completely degraded (Fig. 2a3, b3, and c3). However, the CW structure of the 1-MCP-treated fruits remained on the eighth day, and the middle microfilaments were arranged closely and exhibited intense coloration. The lignin content significantly accumulated in the outer palisade cells and inner pulp compared to the control fruit (Fig. 2a5, b5, and c5). At 11 days after 1-MCP treatment, the cell structure did not disintegrate and the ultrastructure of the CW microfibril structure was still present. Outer primary CW degradation resulted in the damage or even destruction of the nuclear and plasma membranes. The microfilaments were arranged closer together and were darker in color. The lignin accumulation was lower compared to the eighth day, but it was significantly increased compared to the controls (Fig. 2a6, b6, and c6).

### Expression analysis of ethylene signal transduction and *CpMAD1/3* genes

**Fig. 3:**
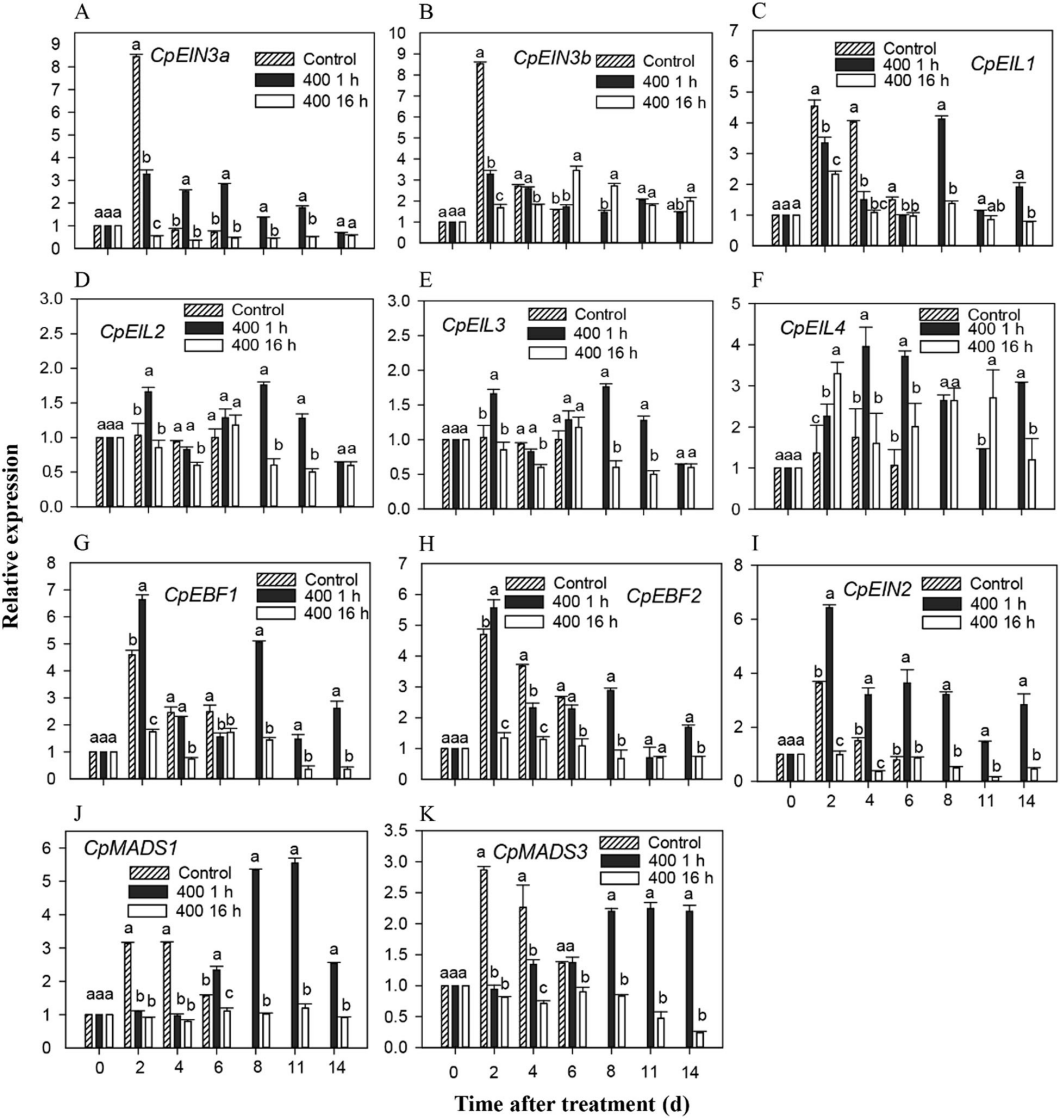
Expression analysis of ethylene signal transduction and *CpMAD1/3* genes. Expression patterns of *CpEIN3a/b* **a**–**b***, CpEIL1/2/3/4* **c**–**f**, *CpEBF1/2* **g**–**h**, *CpEIN2* **I**, and *CpMAD1/3* **j**–**k** after 1-MCP treatment. The expression data at different time points are relative to that of 0 d (untreated fruit), which was set as 1. The data represent the means of three biological replicates (mean ± SD). Different letters between treatments indicate a 5% level of significant difference

Nine ethylene signaling transduction genes and *CpMAD1/3* were isolated, and their expression profiles were assessed by RT-qPCR following 1-MCP treatment. Figure 3 shows that the gene expression was strongly inhibited by the unsuitable 1-MCP treatment.

The *CpEIN3a*, *CpEIL1/2/3, CpEBF1/2*, *CpEIN2*, and *CpMAD1/3* genes were upregulated 1 h after 1-MCP treatment but completely inhibited 16 h after 1-MCP treatment. *CpEIN3a/b* and *CpEIL1* expression dramatically increased and peaked at the second day after treatment, and then decreased to a low level in the control fruit. The *CpEIL1* expression in fruits treated with 1-MCP for 1 h shows two expression peaks, which were significantly lower than the control but increased to a second peak with extended storage. Unsuitable 1-MCP treatment severely inhibited *CpEIN3a/b* and *CpEIL1* expression, which were maintained at a low level throughout storage (Fig. 3a–c). *CpEIL2/3/4* showed a similar expression pattern in the control group that was stably expressed during storage. The 1-MCP treatment for 1 h-induced *CpEIL2/3* expression, but not 1-MCP for 16 h. Both 1-MCP treatments increased the *CpEIL4* transcript abundance (Fig. 3d–f). *CpEBF1/2* and *CpEIN2* showed similar expression patterns in the control fruits, which dramatically increased at the second day and then gradually decreased. The expression of these gene in fruits treated with 1-MCP for 1 h was initially inhibited and then subsequently upregulated during late storage, and the peak values were significantly higher than the controls, but strongly inhibited by 16 h of 1-MCP treatment (Fig. 3g–i). The expression analysis showed that *CpMADS1* and *CpMADS3* have similar expression patterns in the controls, which increased after ethephon treatment, peaked at the second day after treatment, and then gradually decreased. The 1 h 1-MCP treatment delayed *CpMADS1/3* from reaching its peak expression level during storage, but it did not significantly affect the actual peak value. However, the 16-h 1-MCP treatment severely inhibited *CpMADS1* and *CpMADS3* expression (Fig. 3j, k).

### CpEBF1 interacts with the CpMADS1/3 and CpEIL1 proteins

The Y2H method was used to detect gene transcription activation and picked the bait proteins from genes with no transcriptional activation to screen for interacting proteins. CpEBF1 did not show transcriptional activation and was thus chosen as a bait protein (Supplementary Figure 1).

The quality of the cDNA library was checked by electrophoresis, plate growth, and library fragment detection, and it met library standards (Clontech, 634901) (Supplementary Figure 2). We then used CpEBF1 as a bait protein to screen for interacting proteins in the cDNA library.

**Fig. 4:**
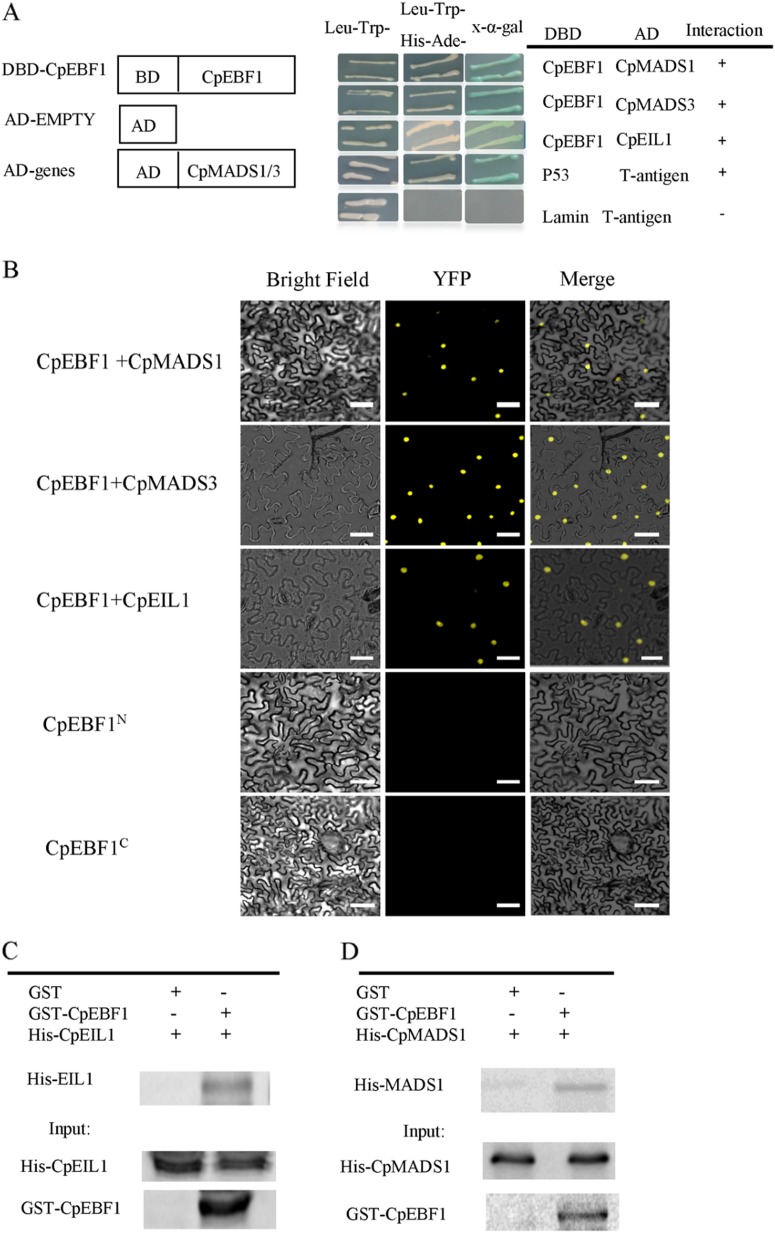
Y2H, BiFC, and GST pull-down assays show that CpEBF1 interacts with the CpMADS1/3 and CpEIL1 proteins. **a** CpEBF1 interacts with the CpMADS1/3 and CpEIL1 proteins as detected using Y2H. CpEBF1 was ligated into the pGBKT7 vector. The CpEBF1 recombinant protein was used as a bait to capture the interaction proteins from the cDNA library. CpMADS1/3 and CpEIL1 were used as prey proteins. Full-length CpMADS1/3 and CpEIL1 were each cloned into pGADT7. The Y2H yeast strains were co-transformed with CpEBF1 + CpMADS1/3 or CpEBF1 + CpEIL1. pGBKT7-53 + pGADT7-T or pGBKT7-lam + pGADT7-T was used as a positive or negative control. Synthetic dropout (SD) medium lacking Trp, Leu, His, and Ade was used for growing the yeast. Blue plaques indicate interactions between two proteins in the presence of chromogenic substrate X-α-gal; **b** BiFC shows the interaction between CpEBF1 with MADS1/3 and CpEIL1 in tobacco leaf epidermal cells. CpEBF1, CpMADS1/3, and CpEIL1 were fused with the N and C termini of YFP. CpEBF1 with either N or C-terminal alone and was used as the negative control. Bars = 50 μM. **c** and **d** The protein interactions were analyzed using a GST pull-down assay. Recombinant GST-tagged CpEBF1 and His-tagged CpMADS1 and CpEIL1 proteins were produced. GST- and His-antibodies were used for western blot analysis. The bands detected by the GST antibody in the pull-down protein sample represent the interacting proteins

Figure 4 shows the results of the Y2H, BiFC, and GST pull-down assays, which confirmed the interaction of CpEBF1, CpMADS1/3, and CpEIL1 in vivo and in vitro. We used CpEBF1 as bait protein to screen the cDNA library, which identified CpMADS1/3 and CpEIL1 (Fig. 4a). The results demonstrated the interactions of CpEBF1, CpMADS1/3, and CpEIL1 in vivo (Fig. 4b). A strong YFP fluorescent signal was observed in the nucleus after the co-transformation of CpEBF1 and CpMADS1/3 or CpEIL1, whereas no YFP fluorescent was detected in CpEBF1-YFP^N^ (Fig. 4b). Finally, the GST pull-down method was used to verify their interactions further. S-transferase-tagged CpEBF1 (EBF1-GST) and histidine-tagged CpMADS1/3 and CpEIL1 were constructed. The fusion proteins were then purified, whereas CpMADS3 did not obtain purified protein. Figure 4c, d shows the confirmation of the interactions between CpEBF1, CpMADS1, and CpEIL1.

**Fig. 5:**
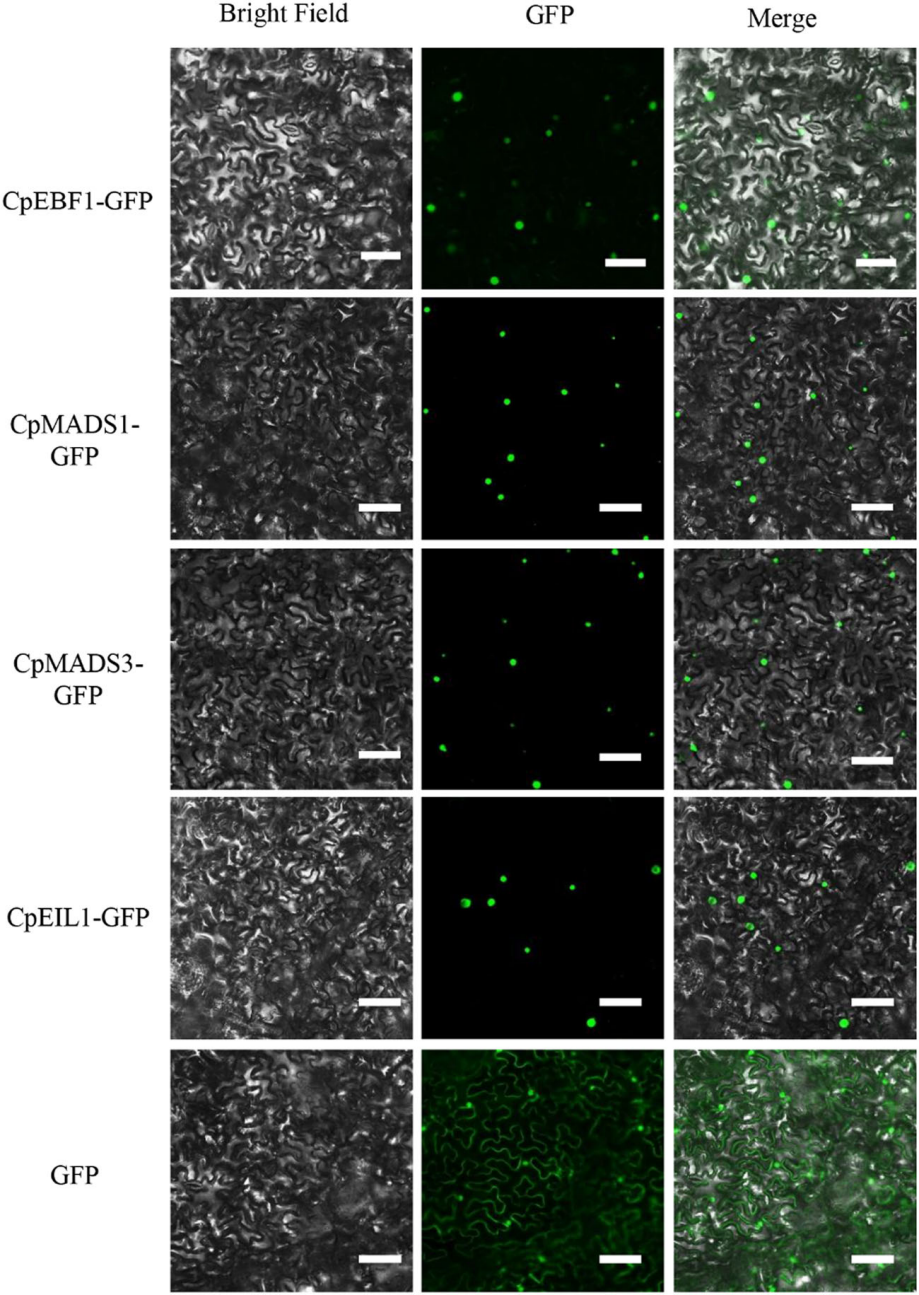
Subcellular localization of CpEBF1, CpMADS1/3, and CpEIL1. All of the pENTR vectors were incubated with the pGWB5 vector together with LR clonase (Invitrogen), resulting in CpEBF1-GFP, CpMADS1/3-GFP, and CpEIL1-GFP fusion proteins. Leaves from 3- to 5-week-old *Nicotiana benthamiana* plants were infiltrated with GV3101 strains containing the pGWB5 recombinant vector. An empty pGWB5 vector that carries free GFP was used as the control. All the fluorescence microscopy observation assays were repeated at least thrice. Bars = 50 μM

The MADS sequences from different species were selected for multiple sequence alignment. Supplementary Figure 3 shows that the CpMADS1/3 proteins contained four conserved domains, namely the M, I, K, and C domains. To identify the portions of CpMADS1/3 that were interacting with CpEBF1, the Y2H yeast strains were co-transformed with DBD-CpEBF1 and AD-(M), AD-(M + I), AD-(M + I + K), and AD-(K + C). The results indicate that CpEBF1 interacts with the K domain of the MADS protein (Supplementary Figure 3). A subcellular localization analysis showed that CpEBF1, CpMADS1, CpMADS3, and CpEIL1 are all located in the nucleus (Fig. 5).

### CpEBF1, CpMADS1/3, and CpEIL1 and their interaction activates the expression of fruit softening-related genes in vivo

**Fig. 6:**
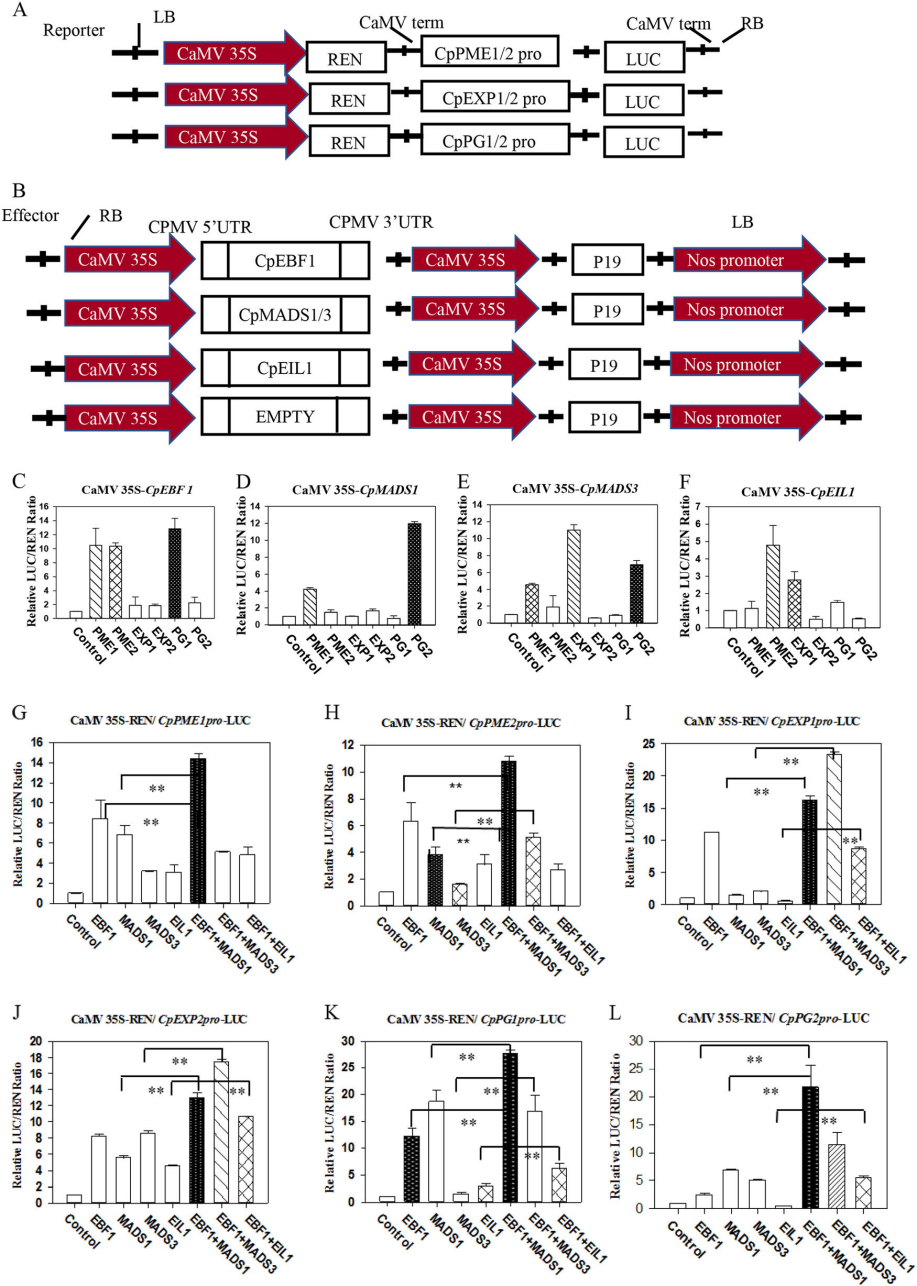
Transient expression assays. The promoters of *CpPME1/2*, *CpEXP1/2*, and *CpPG1/2* were cloned into a pGreenII 0800-LUC double-reporter vector **a**, while CpEBF1, CpMADS1/3, and CpEIL1 were cloned into the pGreenII 62-SK vector as effectors **b**. The LUC activity was normalized to the REN activity (internal control). **c**–**f** Transcriptional activity of CpEBF1 **c**, CpMADS1/3 **d**, **e**, and CpEIL1 **f** on the cell wall degradation-related gene promoter of *CpPME1/2, CpEXP1/2*, and *CpPG1/2*. **g**–**l** Transcriptional activity of CpEBF1, CpMADS1/3, CpEIL1, CpEBF1 + CpMADS1/3, and CpEBF1 + CpEIL1 on the promoters of *CpPME1/2* **g**, **h**, *CpEXP1/2* **i**, **j**, and *CpPG1/2* **k**, **l**. At least six transient assay measurements were performed for each assay. The values represent the means ± SE. Asterisks indicate significantly different values (**P < 0.01)

To determine whether CpEBF1, CpMADS1/3, and EIL1 or their interactions affect the promoter activity of fruit softening-related genes, transient assays were conducted using the dual-luciferase reporter assay. PG, CX, PME, and PL have been previously reported to play important roles in papaya fruit softening. First, the promoters of the fruit softening-related genes were isolated, and sequence analyses were conducted to identify the *cis* regulatory elements. Different putative *cis*-elements were identified, and most of them are related to hormone and stress responses (Supplementary Table 7). Then, the LUC reporter construction under the control of fruit softening-related gene promoters together with an overexpression vector carrying CpEBF1/2, CpMADS1/3, and CpEIL1 under the control of the CaMV35S promoter were co-transformed into the tobacco leaves (Fig. 6a, b). The expression of CpEBF1 alone significantly activated the promoters of the *CpPME1/2* and *CpPG1* genes, whereas no significant effects on the *CpPG2* and *CpEXP1/2* genes were detected (Fig. 6c). Both CpMADS1 and CpMADS3 induced the activities of the *CpPME1* and *CpPG2* promoters. CpMADS3 also significantly induced the activities of the *CpEXP1* promoter (Fig. 6d, e). The expression of CpEIL1 significantly induced the activities of the *CpPME2* and *CpEXP1* promoters (Fig. 6f). When CpEBF1 and the interacting partners of CpMADS1, CpMADS3, and CpEIL1 were co-expressed in the same leaves, the promoter activities for all the CW-degradation-related-genes tested were upregulated compared to their individual effects (Fig. 6g–l). CpEBF1 interactions with CpMADS3 and CpEIL1 enhanced the promoter activity of fruit CW-degradation-related genes.

### Changes in CW-degrading enzymes and expression patterns of CW-degrading genes in papaya

**Fig. 7:**
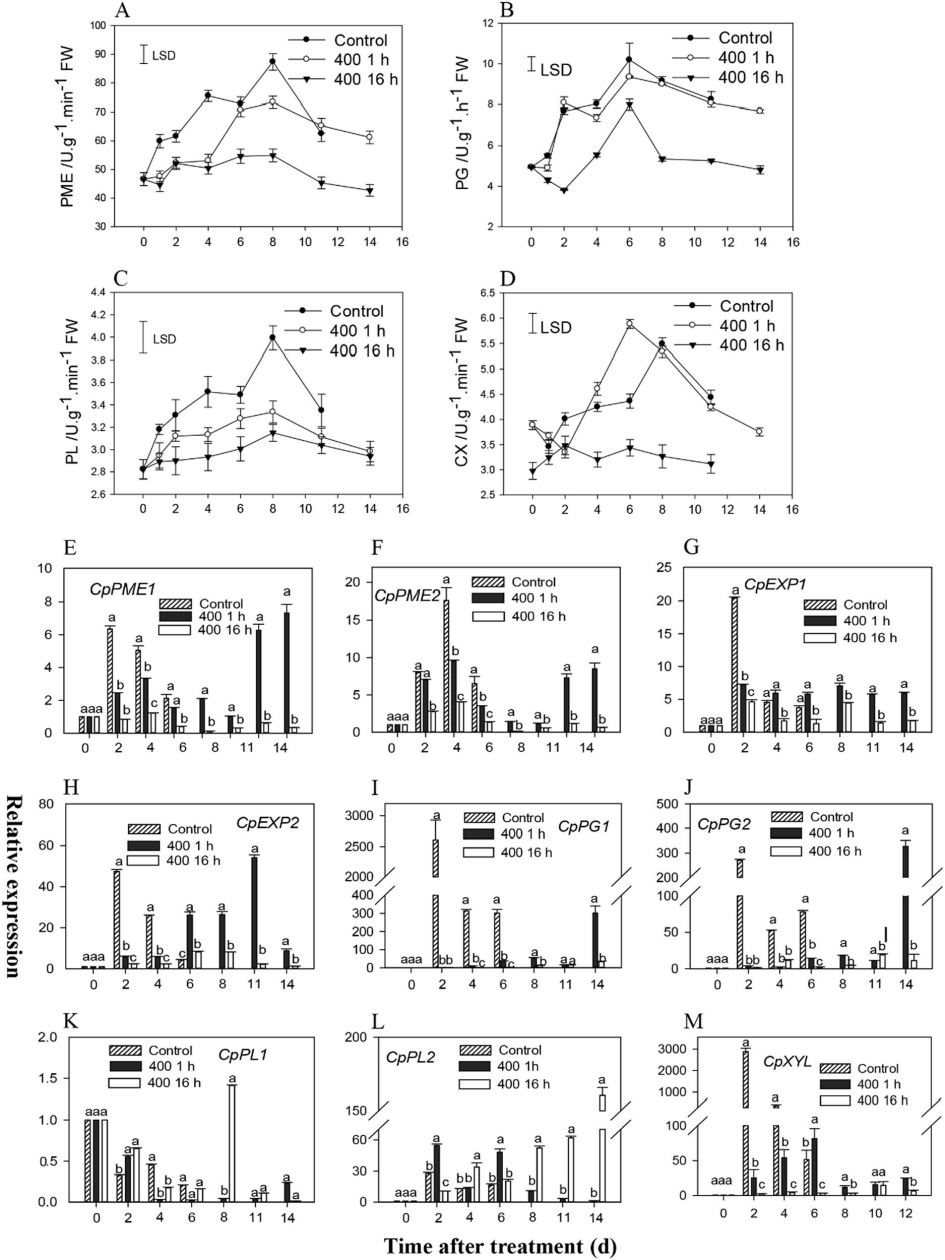
Changes in cell wall-degrading enzymes and expression patterns of cell wall-degrading genes in papayas after 1-MCP treatment. **a**–**d** Cell wall-degrading enzyme activities after 1-MCP treatment. **a** polygalacturonase (PG); **b** cellulose (CX); **c** pectin methylesterase (PME); and **d** polygalacturonic acid (PL). **e**–**p** Expression patterns of cell wall-degrading genes after 1-MCP treatment as indicated by RT-qPCR. Expression analysis of *CpPME1, 2, 3*, and *4* **e**–**h**; *CpPG1* and *2* **i**, **j**; *CpEXP1* and *2* **k**, **l**; *CpPL1* and *2* **m**, **n**; *CpGAL* **o**, and *CpXYL* **p** as conducted by RT-qPCR. The control group only shows the data after 6 days of treatment. The expression data at different time points are relative to that of 0 d (untreated fruits), which was set at 1. The data are the means ± SD of three biological replicates. Different letters between treatments indicate significant differences at the 5% level by one-way ANOVA. The LSD at *P* = 0.05 was calculated to compare the differences between the means of different treatments

Figure 7 shows that PME, PG, PL, and CX have similar activities in relation to ethylene production, which increased during initial storage, peaked, and then subsequently decreased. This pattern is closely related to fruit ripening and softening. The application of 1-MCP for 1 h significantly repressed their activities and delayed the time to reach peak activities (Fig. 7a–d). However, the 16 h-1-MCP treatment severely inhibited the activities of all the enzymes, particularly PME, PL, and CX (Fig. 7a–d). Different genes encoding CW-degrading enzymes were identified, and they included *CpPME1, CpPME2, CpPG1, CpPG2, CpEXP1, CpEXP2, CpPL1, CpPL2*, and *CpXYL*. The expression of most of these genes were strongly induced by ethephon treatment (Fig. 7e–m). The expression of *CpPME1* and *CpPME2* showed similar expression profiles, which strongly increased at the second day and then gradually decreased. The 1 h-1-MCP treatment significantly repressed their transcript levels and delayed the time to reach peak expression levels. The 16 h-1-MCP treatment completely inhibited their expression, which were kept at a very low level during the entire storage period (Fig. 7e, f). *CpPG1* and *CpPG2* were significantly upregulated in the control fruits after ethylene treatment and peaked at the second day, which reached ~2500-fold and 300-fold, respectively, on day 0. In addition, the expression remained at extremely high levels during the entire storage period. The 1 h 1-MCP treatment effectively repressed *CpPG1* and *CpPG2* expression and delayed the time to reach peak levels. However, the 16-h 1-MCP treatment effectively inhibited their expression during the entire storage period (Fig. 7g, h). The expression pattern of *CpEXP1* and *CpEPX2* was similar to that of the *CpPME1/2/3* genes, which increased with fruit ripening and then decreased during extended storage. The 1-h-1-MCP treatment significantly repressed the transcript levels and delayed the time to reach peak levels of expression, but the 16-h 1-MCP treatment completely inhibited their expression (Fig. 7i, j). *CpPL1* and *CpPL2* showed distinct expression patterns. *CpPL1* expression decreased with fruit ripening, and no effects were observed after 1-MCP treatment except on the eighth day, when the 16-h 1-MCP treatment resulted in its upregulation compared to the others. However, *CpPL2* expression significantly increased after ethephon treatment, and then decreased. Both 1-MCP treatments resulted in higher *CpPL2* transcript levels, particularly after the 16-h treatment, which reached a peak that was 150-fold higher than that of day 0 (Fig. 7k, l). The *CpXYL* gene was also strongly induced by the ethephon treatment, which peaked on the second day after the ethephon treatment to ~3000-fold higher than that of day 0. However, both 1-MCP treatments significantly inhibited *CpXYL* expression (Fig. 7 m). These results coincide with their enzymatic activities and pulp hardness phenotype.

## Discussion

The fruit firmness is very important for the fruit quality, and the CW is a key factor for maintaining the fruit firmness. The CW is a complex structure that consists of pectin, protein, cellulose, and hemi-cellulose. The CW composition is rapidly degraded with the fruit ripening in the controls (Fig. 2). However, the CW structure in the 16-h 1-MCP treatment remained intact, the organelle structure remained clear, and the cells were arranged in an orderly and tight manner-throughout storage. The intact CW structure helps to maintain cellular strength and toughness, which in turn maintains flesh firmness. The thinning of the CW is due to the roles of the cell wall-degrading enzymes on the CW polysaccharide structure, causing a loss in pulp tissue rigidity. The primary CW of fruits is primarily comprising of polysaccharides (pectin, cellulose, and hemi-cellulose)^39^. During the ripening of papaya fruit, the pectin, cellulose, and hemicellulose depolymerization increase^40^. Lignin is a complex aromatic polymer that is deposited on the secondary CW of all vascular plants^41^. Lignin is tightly connected to other CW components and can therefore be regarded as a “cellular glue” that provides strength to plant tissues and fibers as well as rigidity to the CW^42^. During extended storage, lignin with increased PAL, CAD, and POD enzymatic activity was determined in 1-MCP treated fruits, revealing that 1-MCP enhances the accumulation of lignin in the fruits, particularly the core tissues^43^. By contrast, 1-MCP inhibited the activities of PAL, CAD, and POD, as well as lignin accumulation in post-harvest shoots, and decreased lignin accumulation in loquat fruits^44^. In our study, 1-MCP promoted the accumulation of lignin to maintain the higher hardness of papaya fruit (Fig. 2). Phytase does not degrade lignin because of its complex structure in higher plants^45^. Different plants and parts of the same plant have various types of lignin. The enhancement of lignin biosynthesis due to biotic stress has been attributed to the stimulation of the amphetamine metabolism pathway as well as the induction of extracellular body lignin polymerization^46^. The complexity of the lignin accumulation needs further in-depth study. Paraffin sectioning showed that 1-MCP inhibits cellulose degradation to maintain fruit hardness (Fig. 2). Fruit softening disorders caused by 1-MCP treatment might be due to the failure to degrade CW cellulose microfilaments and the accumulation of lignin.

Ethylene plays critical roles in fruit ripening^3^. Ethylene signal transduction has been studied extensively in plant development and fruit ripening^14^. Two F-box proteins, EBF1, and EBF2, regulate the stability of EIN3 through the ubiquitin/26S proteasomal degradation pathway, and they play critical roles in ethylene signal transduction in *Arabidopsis* ^13^. The analysis of mutant combinations showed that both EIN3 and EIL1 are the primary targets of EBF1/2^47^. EBF1 and EBF2 promote the more rapid recovery in ethylene responses after the ethylene levels dissipate^48^. Eighteen deletion mutations in EBF1/EBF2 stabilize EIN3 and enhance ethylene responses; whereas, the overexpression of *EBF1* or *EBF2* results in ethylene insensitivity, indicating that EBF1 and EBF2 negatively regulate ethylene responses^49^. No differences between the tomato plants silenced for any of the *Sl-EBF* genes and the control plants were observed, indicating the functional redundancy of the *Sl-EBF* genes^14^. However, studies on *EBF* genes have primarily focused on their roles in regulating ethylene responses in *Arabidopsis* and tomato, and the present study uncovered their roles in modulating fruit ripening in papaya. In the present study, the expression of *CpEBF1/2* was strongly induced by ethephon treatment but inhibited by long-term 1-MCP treatment (400 nL L^−1^, 16 h) and it was initially inhibited during storage by suitable 1-MCP treatment (400 nL L^−1^, 1 h) (Fig. 3g, h). It seems that *CpEBF1/2* are positively related to the ethylene responses and fruit ripening. However, further functional characterization is needed to elucidate the roles of *CpEBF1/2* in fruit ripening. In *Arabidopsis*, EIN3/EILs can feedback activate the EBF1/2 promoter and promote its expression^47^. We hypothesize that long-term 1-MCP treatment inhibits the expression of *CpEIN3/EIL*s (Fig. 3a–f), which feedback inhibits *CpEBF1/2* gene expression. However, silencing of the *EBF1/2* genes in *Arabidopsis* and tomatoes showed high sensitivity to ethylene because the EIN3/EILs were not inhibited, and the EIN3/EILs were further stabilized by the reduction in degraded enzymes. Our results also showed the complexity of *CpEBF1/2* in regulating fruit ripening in ethylene signaling transduction.

The MADSs gene family is closely related to fruit ripening and senescence in many plants^21^, but it has not been reported in papaya. Members of the *MADSs* gene family, including *SlMADS-RIN*^22^, *FRUIFULL*^50^, and *FYFL*^51^, are important for fruit expansion and ripening. In the present study, *CpMADS1/3* was strongly induced by ethylene, but it was significantly inhibited by 1-MCP treatment (Fig. 3j, k), which may positively regulate papaya fruit ripening. Similar *MADS-box* genes involved in ripening have been described in other fruits, such as bananas^19^, tomatoes^22^, and apples^52^. The *MaMADS3* expression was repressed in peels of both *MaMADS1* and *MaMADS2*-repressed lines because of the reduction in ethylene production in bananas^19^. A banana MADS-box gene was obtained by cDNA microarray, showing that it is upregulated during the early stages of banana ripening^53^. Tomato *SlMADS-RIN* plays an ethylene-independent role in ripening downstream from the ethylene action, as the exogenous application of ethylene to *rin* tomatoes did not restore fruit ripening^22^. In contrast to these results, silencing the *MADS1* gene leads to the early ripening of tomato fruits, indicating that it plays a negative regulatory role in tomatoes^22^. SLTAGL1 does not respond to ethylene, suggesting that MADS has various regulatory patterns in different plants^18^. Thus, *CpMADS1/3* may act as an important player in papaya fruit ripening and softening.

MADS-box genes act upstream of the ethylene pathway and primarily regulate ethylene synthesis to control fruit ripening^22,23^. No study has shown that MADS interacts with the ethylene signal transduction components in plants. In the present work, we showed that CpEBF1 interacts with CpMADS1/3, these proteins are closely related to papaya fruit ripening and fruit softening (Supplementary Tables 8-10), and their expression was severely inhibited by unsuitable 1-MCP treatment, which caused the fruit softening disorder. A phylogenetic analysis showed that CpMADS1 is closely related to MaMADS2/4 in bananas and LeMADS-RIN in tomatoes. CpMADS3 is closely related to AtAGL6 from *Arabidopsis* (Supplementary Figure 3). The peak values of *CpEBF1* and *CpMADS1/3* expression in normally ripening fruits are exactly the same as the values when the hardness begins to decline rapidly (Supplementary Figure 4). We hypothesize that CpEBF1 and the interacting proteins CpMADS1/3 may play important roles in fruit softening. Figure 6 shows that CpEBF1 and the interacting CpMADS1/3 and CpEIL1 proteins could activate the promoter activities of important CW-degradation genes. Importantly, the interactions between CpEBF1 and CpMADS1 and CpMADS3, and CpEIL1 could enhance the activation effects on the promoters of CW-degradation genes. These results indicate that CpEBF1 not only participates in the ethylene signal transduction pathway to regulate papaya fruit ripening and softening but also in direct regulation of the papaya fruit softening process by interacting with CpMADS1, CpMADS3, and CpEIL1. It has also been reported that ethylene-signaling genes regulate the expression of softening genes to affect softening in many fruits. In kiwifruits, *AdERF9* is involved in regulating the fruit ripening processes by suppressing *AdXET5* promoter activity.^54^. The other two transcription factors, *AdEIL2* and *AdEIL3*, could also activate the transcription of the ripening-related genes *AdXET5* and *AdACO1*^54^. In bananas, the EAR-motif-containing *MaERF11* can bind to the promoters of *MaACO1, MaACS1*, and *MaEXPs* and repress their activities to regulate fruit ripening^55^. Papaya *CpERF9* regulates fruit ripening by binding directly to the *CpPG5* and *CpPME1/2* promoters, causing the transcriptional repression of CW-modification genes^28^. In addition, MADS regulated the expression of genes related to fruit softening, thereby influence the softening of fruits. A similar site sequence for most MADS box protein binding is the CArG-box, or CC (A/T) GG. The MADS-RIN gene in tomatoes binds to the CArG-box of *ACS2*, *ACS4*, *TBG4*, *EXP1* and *PG* to regulate fruit ripening and softening^56^. In papayas, CpMADS1 or CpMADS3 might also directly bind to the promoters of *CpEXPs* or *CpPGs* to regulate fruit softening. However, the MADS binding site CArG-box was not found in the promoters in *CpEXP1/2* or *CpPG1/2*, which we isolated. There may be other binding sites in the CW-degrading genes that bind to CpMADS1/3, or other un-isolated CW gene promoters that contain the CArG-box site. These hypotheses may explain the interaction of CpEBF1 and CpMADS1/3, which enhances the transcriptional activity of CW-degrading genes and promotes papaya softening. In the present work, several CW-degradation-related genes were selected, including *CpPME1/3, CpEXP1/2, CpPG1/2, CpPL1/2*, and *CpXYL*. Our results are consistent with previous research in that 1-MCP treatment inhibits the expression of most of these genes as well as the corresponding enzymatic activities. Notably, unsuitable 1-MCP treatment almost completely inhibited the expression of these genes and the enzyme activities (Fig. 7). Among the CW-degrading-related genes, *CpPGs, CpGAL, CpPMEs*, and *CpXYL* are pectinolytic enzymes, which play important roles in CW solubilization during fruit ripening. CpMADS1/3, CpEBF1, and their interactions could significantly activate the promoter activities of these CW-degrading genes and further promote fruit ripening. These results indicate that MADS not only acts as an important player upstream of the ethylene pathway but also within and downstream of the ethylene signal transduction pathway, which greatly improves our understanding of the function of MADS in plants.

Based on the results of previous studies and the present investigation, we propose that CpEBF1 plays dual roles in papaya fruit ripening. First, CpEBF1 acts negatively on the ethylene signal transduction pathway by degrading the CpEIN3 protein and thus delaying fruit ripening. However, the interaction of CpEBF1 with CpMADS1/3 resulted in a lower amount of free CpEBF1 to degrade CpEIN3/EIL1, which helps to stabilize CpEIN3/EIL1, enhances ethylene responses and promotes fruit ripening. Second and more importantly, CpEBF1, CpMADS1/3, and their interactions can activate the promoters of fruit softening-related genes, which directly promote the expression of softening-related genes and enzyme activities.

**Fig. 8:**
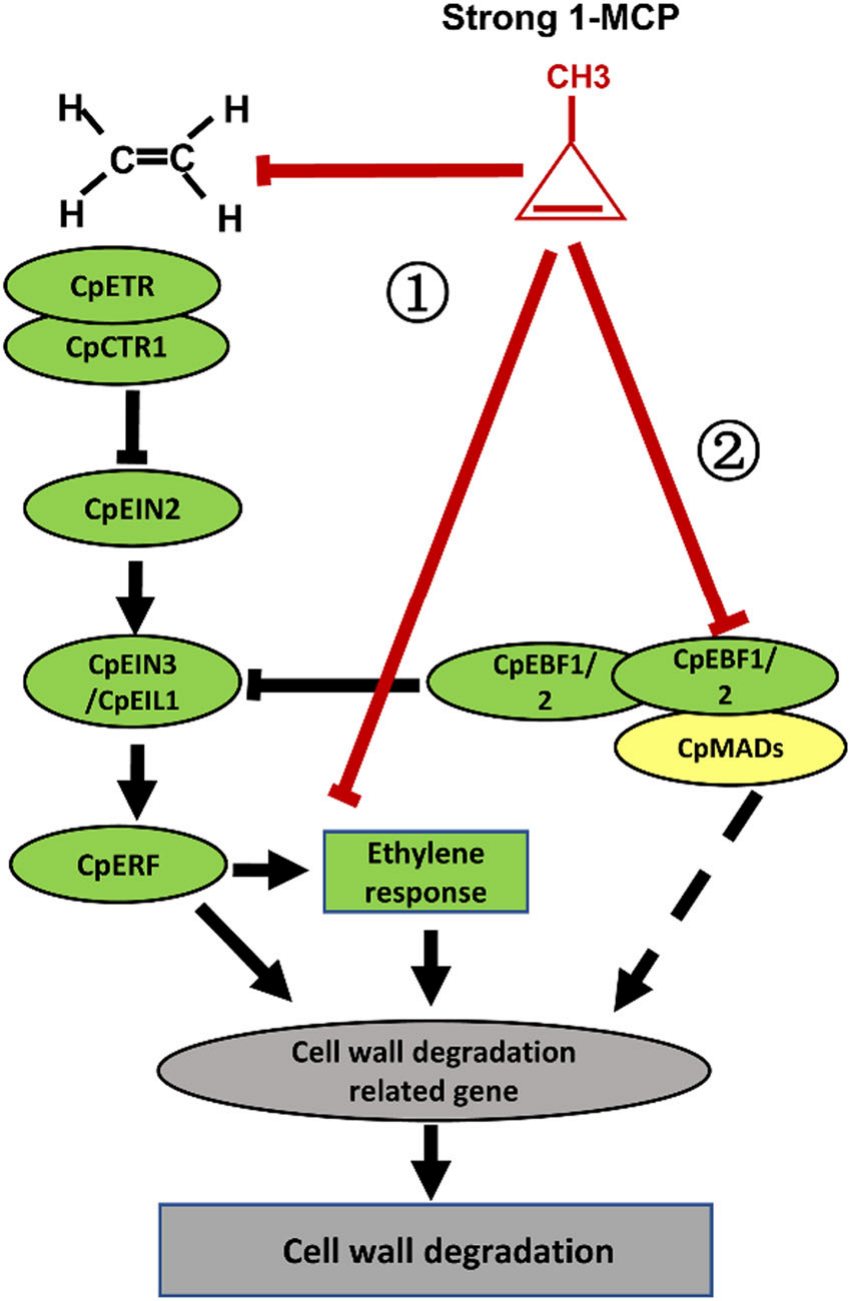
Putative model for the effect of 1-MCP treatment on fruit softening. Fruit softening is due to the different effects of ethylene activity. Ethylene responses play a major role in fruit softening. Several elements in the ethylene signal transduction pathway can act directly on cell wall degradation-related genes, such as *ERF*s and *EBF*s, triggering fruit cell wall degradation. The failure of fruit softening may be due to the severe inhibition of *CpEBF1* and *CpMADS1/3* expression by unsuitable 1-MCP treatment (400 nL L^−1^, 16 h). First, unsuitable 1-MCP treatment severely inhibits ethylene production and the expression of most genes in the ethylene signal transduction pathway, which significantly reduces ethylene responses and the ethylene effect on fruit ripening and softening. Second, unsuitable 1-MCP treatment completely inhibits the expression of *CpEBF1* and *CpMADS1/3*, which may also reduce the interaction between CpMADS1/3 and CpEBF1, thereby preventing the direct activation of fruit softening genes and enzyme activities

In taking these findings together, we propose a model for the roles of CpEBF1, CpMADS1/3, and their interaction in regulating papaya fruit ripening (Fig. 8). Fruit softening is the result of different effects of ethylene. Downstream ethylene responses play a major role in fruit softening. Several elements in the ethylene signal transduction pathway can also act directly on CW-degradation-related genes, such as *ERF*s and *EBF*s, triggering fruit CW degradation. Suitable 1-MCP treatment reduces ethylene production and represses the expression of most genes in the ethylene signal transduction pathway, and it reduces the ethylene responses and effects on fruit ripening and softening, which could delay fruit ripening and softening. The papaya softening disorders caused by unsuitable 1-MCP treatment may be due to the severe inhibition of *CpEBF1* and *CpMADS1/3* expression (Fig. 8). First, unsuitable 1-MCP treatment severely inhibited the expression of most genes involved in the ethylene signal transduction pathway, as well as reduced the ethylene production, which significantly decreased the ethylene responses and effects on fruit ripening and softening. Second, unsuitable 1-MCP treatment completely inhibits the expression of *CpMADS1/3* and *CpEBF1*, which could reduce the interaction between CpMADS1/3 and CpEBF1, thereby preventing the direct activation of fruit softening genes.

## Electronic supplementary material

supplemetary figure1-4

supplemetary figure1-3

supplemetary table1-10
