# Supplementary material for: The interaction of CpEBF1 with CpMADSs is involved in cell wall degradation during papaya fruit ripening
Source: Hortic Res. 2019 Jan 1;6:13. doi: 10.1038/s41438-018-0095-1 (PMC6312555; doi:10.1038/s41438-018-0095-1)
Supplement: Supplementary file 2 — supplemetary figure1-3 [file 41438_2018_95_MOESM2_ESM.pdf]

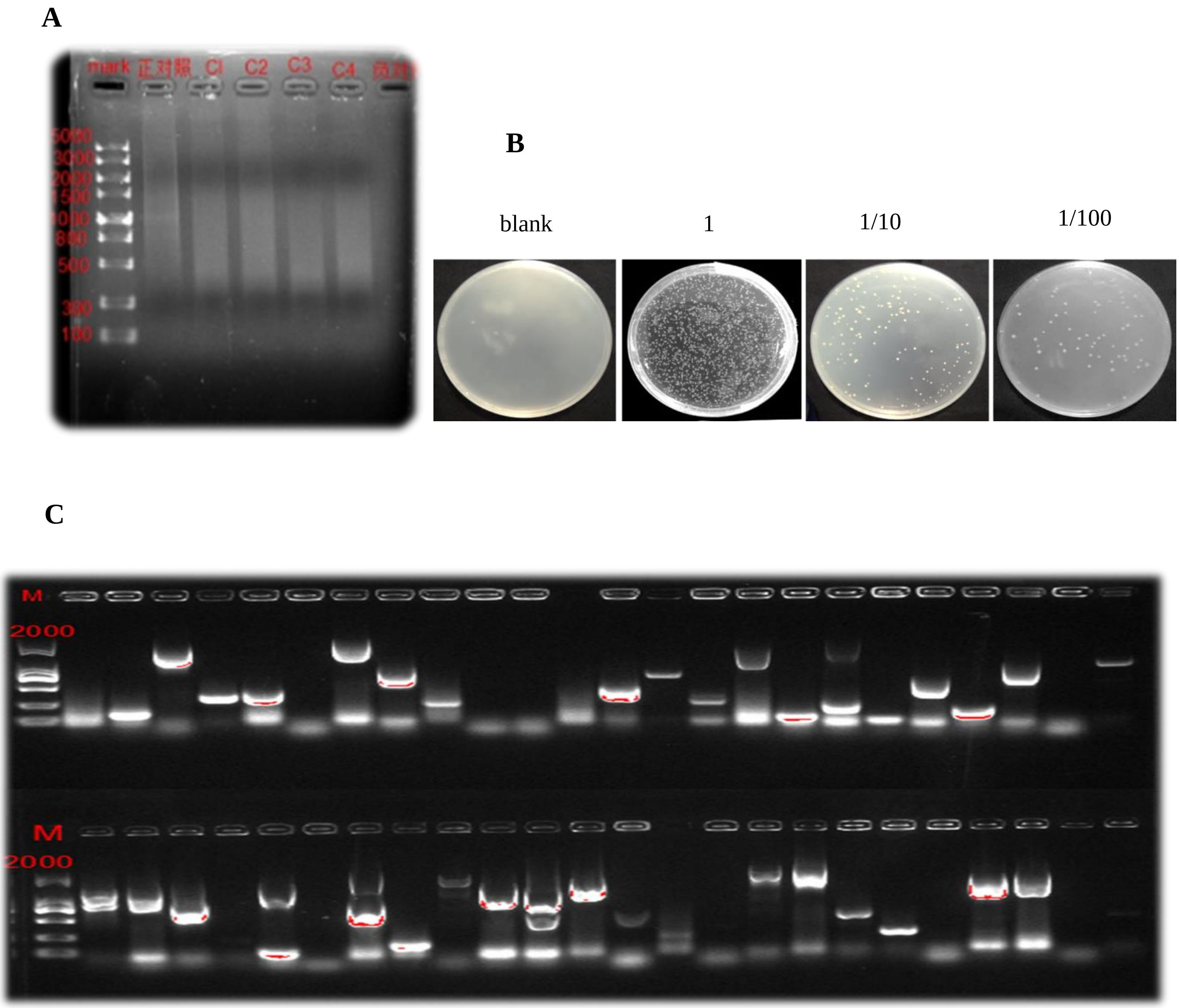

Supplementary Figure 1. cDNA library construction and quality testing. A, Electropherogram Detection of CDNA Library. M represent mark 5000, L1was a positive control and represented the mouse cDNA library from Takara. L2-4 are the experimental group, representing a different number of cycles amplified papaya cDNA library, the number of cycles were 20,21,22. L5 was negative control only reagent without cDNA sample. B, the cDNA library was co-transformed into the Y187 strain with the pGADT7-rec vector. The yeast cells to grow on the synthetic medium lacking Leu and to diluted 10 times, 100 times respectively. C, cDNA library inserted fragment quality testing.

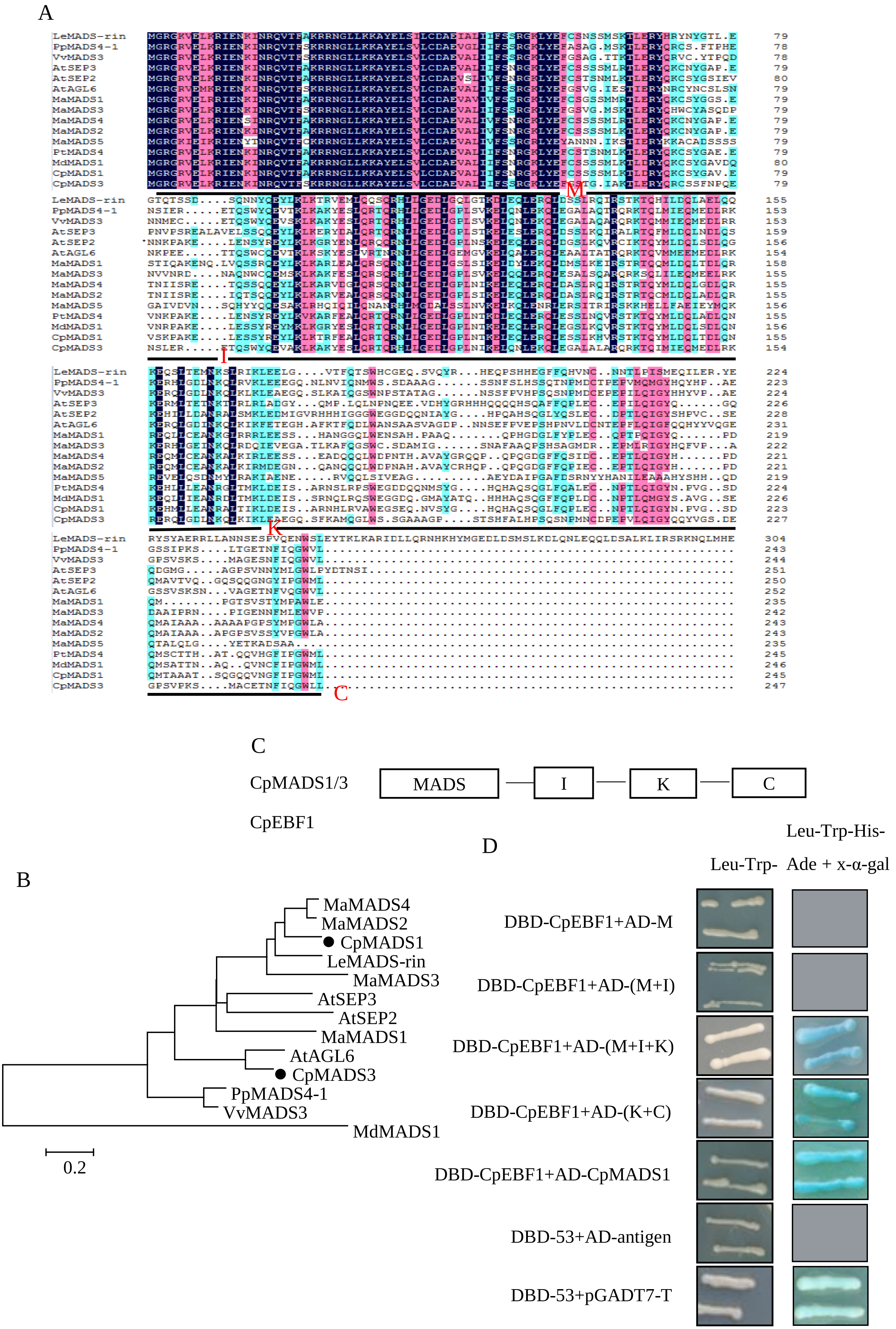
A
LeMADS-rin
PpMADS4-1
VvMADS3
AtSEP3
AtSEP2
AtAGL6
MaMADS1
MaMADS3
MaMADS4
MaMADS2
MaMADS5
PtMADS4
MdMADS1
CpMADS1
CpMADS3
M
I
K
C
C
CpMADS1/3
CpEBF1
MADS
I
K
C
B
MaMADS4
MaMADS2
CpMADS1
LeMADS-rin
MaMADS3
AtSEP3
AtSEP2
MaMADS1
AtAGL6
CpMADS3
PpMADS4-1
VvMADS3
MdMADS1
0.2
D
Leu-Trp-
Leu-Trp-His-
Ade + x-α-gal
DBD-CpEBF1+AD-M
DBD-CpEBF1+AD-(M+I)
DBD-CpEBF1+AD-(M+I+K)
DBD-CpEBF1+AD-(K+C)
DBD-CpEBF1+AD-CpMADS1
DBD-53+AD-antigen
DBD-53+pGADT7-T

Supplementary Figure 2. CpMADS1/3 proteins alignment, phylogenetic analysis, and the domain interacting with CpEBF1. A and B, MADS protein alignment of phylogenetic analysis and between CpMADS1/3 and other plant species from NCBI (Detail information are available in Supplementary Table 4). The evolutionary tree analysis using homologous genes from other species. The protein alignment diagram was drawn by DNAMAN software (A) and the phylogenetic tree was drawn using MEGA 5.0 (B). C and D, CpMADS1/3 protein contained M, I, K, and C domains. CpMADS1/3 was divided into four fragments, and their interaction with CpEBF1 using Y_2H assay. CpMADS1/3 fragments were ligated into the pGADT7 vector and CpEBF1 into the pGBKT7 vector. SD medium for yeast growth lacking Trp, Leu, His and Ade. Blue plaques indicate interaction between two proteins in the presence of chromogenic substrate X-α-gal.

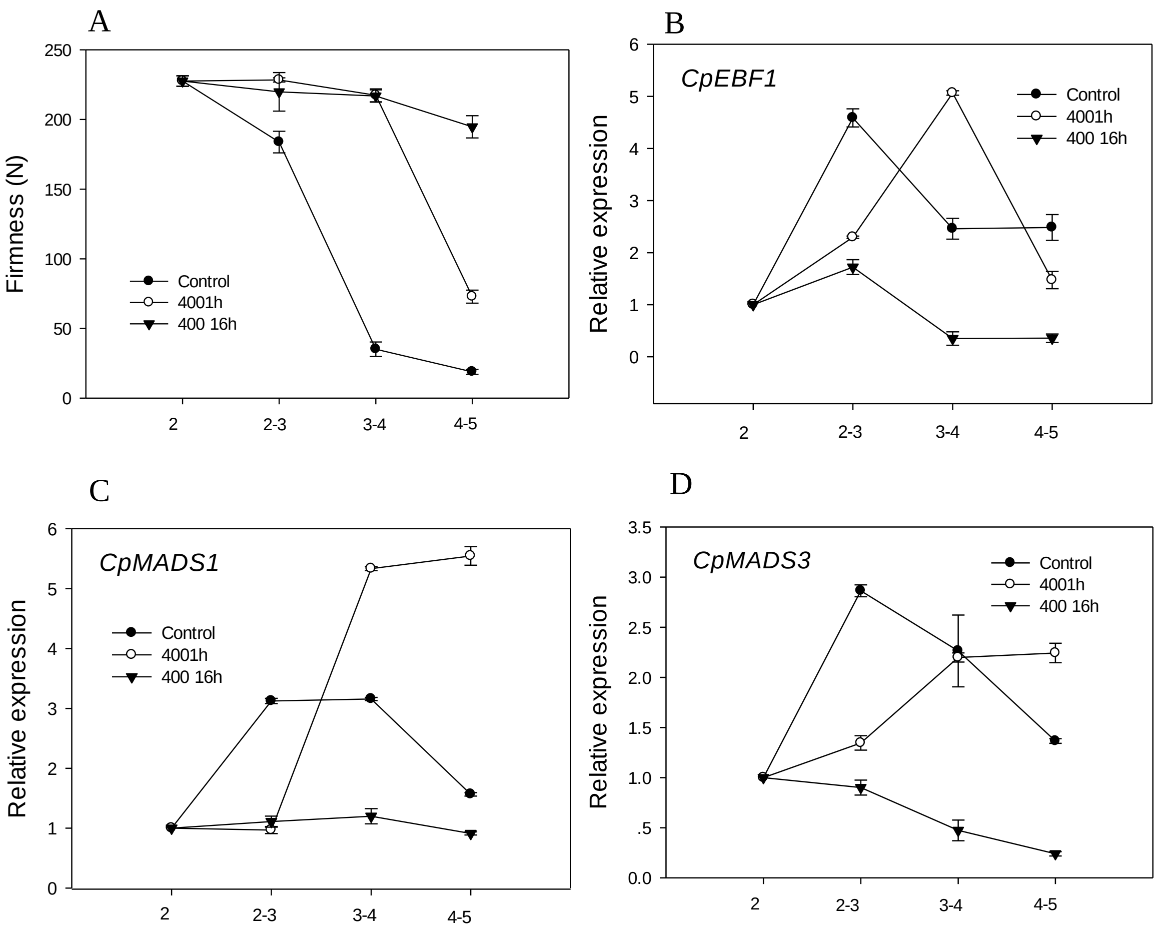

Supplementary Figure 3. The relationship between the coloring index and hardness (A), *CpEBF1* (B), *CpMADS1* (C) and *CpMADS3* (D) gene expression. 2-3: peel coloring index transition from grade 2 to 3 critical point; 3-4 peel coloring index from grade 3 to 4 critical point; 4-5: peel coloring index from grade 4 to 5 critical point.
