# Supplementary material for: The interaction of CpEBF1 with CpMADSs is involved in cell wall degradation during papaya fruit ripening
Source: Hortic Res. 2019 Jan 1;6:13. doi: 10.1038/s41438-018-0095-1 (PMC6312555; doi:10.1038/s41438-018-0095-1)
Supplement: Supplementary file 3 — supplemetary table1-10 [file 41438_2018_95_MOESM3_ESM.pdf]

Supplementary Table 1 Primers used for RT-qPCR

Primer name	Accession	Primer (5'-3')
CpMADS1-F	EU659990.1	CTGGGAGTGAAGGACCTGAA
CpMADS1-R		CCTCTTTCAAGGCGCAATAG
CpMADS3-F	EU659992.1	GAAGGAGGAGATGCTGATGG
CpMADS3-R		CGGGAGAAAGGAATGTTGTG
CpEIN2-F	KF709946	CTCATCAACACAGGTCAAG
CpEIN2-R		TAGCAGCATAGCAGAAGTT
CpEIL1-F	KF709947	CAGCATCACCAGGACCAA
CpEIL1-R		AAGCCAAATCAAAGGGAGAC
CpEIL2-F	evm.model.supercontig_103.60	ATGAAGAGATTGATGTGGAC
CpEIL2-R		GATTACGTTG TGACACTAGG AG
CpEIL3-F	evm.TU.supercontig_84.104	ATTCAACCAGTGGATGCA
CpEIL3-R		GTACGTGT ACGACTCAACTGT
CpEIL4-F	evm.model.supercontig_20.234	AGATGGCTAGAGCTCAAG
CpEIL4-R		CTTATCGGGGAGGAACCACCGGT
CpEIN3a-F	KF709948	GGCTCAACAGTCAGATGG
CpEIN3a-R		CTCCAACAATCTCATTCTCA
CpEIN3b-F	KF709949	CATCACCAGGACCAATACTTC
CpEIN3b-R		GTGTCCATCCTACTGCTG
CpEBF1-F	KF709950	TGCTTAGTCCCTTTCCATC
CpEBF1-R		GCTCACACTCTCCAGAAG
CpEBF2-F	KF709951	GACACAGCCATTCTATTACCTT
CpEBF2-R		CTCAGGGAAACACGACCAT
CpERF1-F	JX532988	TCTCCGCCGTCTGATGATT
CpERF1-R		GCAATATCCAGTTTCACACCAT
CpERF2-F	JX532989	ATGTCTCTCTTCGCTTCA
CpERF2-R		CCCATCTCTTCACTATCC
CpERF3-F	JX532990	GAAGAGGAGGAGGATGGTGAT
CpERF3-R		CAGTTAAGCCACTCTTATCTATCC
CpERF4-F	JX532991	GAGAGCAAGATAATACAGGC

CpERF4-R		GTTAGGGAGAAAAACAAAAA
CpEIF-F	FJ644949.1	AGGCAGGCAAGAGAAGAT
CpEIF-R		TTCATACCGAGTAGCGATTC
CpTBP1-F	JQ678780	GGTAGTAGTAGTTAGGTATGTG
CpTBP1-R		GGCAATCTGGTCTCACT
CpPG1-F	evm.model.supercontig_250.6	TGGTGGTGCGTATAGATGGA
CpPG1-R		ACAAAACCCAGTACCCACCA
CpPG2-F	evm.model.supercontig_92.36	TCCTGAAGCTCACCCTTCAT
CpPG2-R		CCTCAATGCCTTTGAAGCTC
Cp-β-GAL-F	evm.model.supercontig_93.34	GTGCTTGCAACTATGCTGGA
Cp-β-GAL-R		ATAGGTTCGCAGTTGGGTTG
CpARF-F	evm.model.supercontig_33.202	AGGTGGCTGTTTTGTTGAGG
CpARF-R		TCTCTT CCCAAGGTCCAATG
CpXYL-F	evm.model.supercontig_106.45	GCTTCCGCTGTGTTTTATGG
CpXYL-R		ATGATTGGATCGACCTCAGC
CpPL1-F	evm.TU.supercontig_33.89	TCTTCATCTTCAGCCGTTCAAG
CpPL1-R		ACCGCCAGCAATCGTCAA
CpPL2-F	evm.TU.supercontig_4.36	ACCATTAACAGTCAAGGCAACA
CpPL2-R		CCAGTTCTTCCATTCGCTCTC
CpPME1-F	evm.model.supercontig_9.24	TCTTCGGCATCTCCTACTTCC
CpPME1-R		AGTACTGGTACAGAAGTGGATCTC
CpPME2-F	evm.model.supercontig_151.19	GGTCGAACACGAGTGGTAGAGT
CpPME2-R		TCGGCGAAATAAGGAATTTCAGT
CpPME3-F	evm.model.supercontig_27.190	CTTCTTCATCTTCGTGGTGCTG
CpPME3-R		GCTGGCTGTGGCATTAAGGTA
CpPME4-F	evm.model.supercontig_9.25	GCCGTTGATAGATTCGACCTG
CpPME4-R		GAAACGCTTCGGTAGCCTGA
CpEXP1-F	evm.model.supercontig_2.303	GCTGTGGTTCTTGCTACGAA
CpEXP1-R		TGCTGGAGAGGAGGATTGC
CpEXP2-F	evm.model.supercontig_1.419	GTGATGCGTCTGGAACTATGG
CpEXP2-R		TGGCGGTGATGAAGATGGAA

Supplementary Table 2 Primers used for Y_2H

Primer name	Primer (5'-3')	Restriction Site
CpEBF1-BD-F	ATGGCCATGGAGGCCgaattcATGCCTGCTCTTGTCAATTATAATACT	EcoRI
CpEBF1-BD-R	ATGCGGCCGCTGCAggtcgacTCACACCTCCACAAGCTCTCCA	SalI
CpEBF2-BD-F	ATGGCCATGGAGGCCgaattcATGTCAAAGCTCTTTGGATTCACTG	EcoRI
CpEBF2-BD-R	ATGCGGCCGCTGCAggtcgacGGAAAGGATGTCACATCTCCATAGC	SalI
CpEIL1-AD-F	GCCATGGAGGCCAGTgaattcATGATGATGTTTGATGATATGGGACC	EcoRI
CpEIL1-AD-R	CAGCTCGAGCTCGATggatccCTGGAACCAGATCGAAACATCC	BamHI
CpMADS1-AD-F	GCCATGGAGGCCAGTgaattcATGGGGAGAGGAAGAGTAGAGTTG	EcoRI
CpMADS1-AD-R	CAGCTCGAGCTCGATggatccAAGCATCCAGCCAGGGATG	BamHI
CpMADS3-AD-F	GCCATGGAGGCCAGTgaattcATGGGGAGAGGAAGAGTGGAA	EcoRI
CpMADS3-AD-R	CAGCTCGAGCTCGATggatccAAGGAGCCATCCCTGGATGA	BamHI

Supplementary Table 3 Primers used for Pull-down

Primer name	Primer (5'-3')	Restriction Site
CpEBF1-GST-F	TTTCAGGGTCCAGCAggatccATGCCTGCTCTTGTCAATTATAATACTG	BamHI
CpEBF1-GST-R	ATGGTGGCTAGCGCTgaattcGAAAAGAATATCACACCTCCACAAGCT	EcoRI
CpEIL1-His-F	CAGCAAATGGGTCGCggatccATGATGATGTTTGATGATATGGGACC	BamHI
CpEIL1-His-R	TGCGGCCGCAAGCTTgtcgacCTGGAACCAGATCGAAACATCC	SalI
CpMADS1-His-F	CAGCAAATGGGTCGCggatccATGGGGAGAGGAAGAGTAGAGTTG	BamHI
CpMADS1-His-R	TGCGGCCGCAAGCTTgtcgacAAGCATCCAGCCAGGGATG	SalI
CpMADS3-His-F	CAGCAAATGGGTCGCggatccATGGGGAGAGGAAGAGTGGAA	BamHI
CpMADS3-His-R	TGCGGCCGCAAGCTTgtcgacAAGGAGCCATCCCTGGATGA	SalI

Supplementary Table 4 MADS proteins used for phylogenetic tree

Species	Protein	Identifier	Source
Pyrus pyrifolia	PpMADS4-1	AB623160.2	https://www.ncbi.nlm.nih.gov
Vitis vinifera	VvMADS3	AF373602.1	https://www.ncbi.nlm.nih.gov
Arabidopsis	AtSEP3	NM_102272.4	https://www.ncbi.nlm.nih.gov
Arabidopsis	AtSEP2	AF133717.1	https://www.ncbi.nlm.nih.gov
Arabidopsis	AtAGL6	M55554.1	https://www.ncbi.nlm.nih.gov
Musa acuminata	MaMADS3	EU869308.1	https://www.ncbi.nlm.nih.gov
Musa acuminata	MaMADS5	EU869310.1	https://www.ncbi.nlm.nih.gov
Pinus tabuliformis	PtMADS4	KJ711077.1	https://www.ncbi.nlm.nih.gov
Malus domestica	MdMADS1	U78947.1	https://www.ncbi.nlm.nih.gov
Lycopersicon esculentum	LeMADS-rin	NM_001247741.2	https://www.ncbi.nlm.nih.gov
Carica papaya	CpMADS1	EU659990.1	https://www.ncbi.nlm.nih.gov
Carica papaya	CpMADS3	EU659992.1	https://www.ncbi.nlm.nih.gov

Supplementary Table 5 Primers used for Subcellular localization

Primer name	Primer (5'-3')
CpEBF1-PENTR-F	CACCATGCCTGCTCTTGTCAATTATAATACT
CpEBF1-PENTR-R	TCACACCTCCACAAGCTCTCCA
CpEBF2-PENTR-F	CACCATGTCAAAGCTCTTTGGATTCACTG
CpEBF2-PENTR-R	GGAAAGGATGTCACATCTCCATAGC
CpEIL1-PENTR-F	CACCATGATGATGTTTGATGATATGGGACC
CpEIL1-PENTR-R	CTGGAACCAGATCGAAACATCC
CpMADS1-PENTR-F	CACCATGGGGAGAGGAAGAGTAGAGTTG
CpMADS1-PENTR-R	AAGCATCCAGCCAGGGATG
CpMADS3-PENTR-F	CACCATGGGGAGAGGAAGAGTGGAA
CpMADS3-PENTR-R	AAGGAGCCATCCCTGGATGA

Supplementary Table 6 Primers used for Transient expression

Primer name	primer (5'-3')	Restriction Site
pGreenII 62-SK-EBF1-F	CGCTCTAGAACTAGTggatccATGCCTGCTCTTGTCAATTATAATACT	BamHI
pGreenII 62-SK-EBF1-R	GATAAGCTTGATATCgaattcCACCTCCACAAGCTCTCCACG	EcoRI
pGreenII 62-SK-EIL1-F	CGCTCTAGAACTAGTggatccATGATGATGTTTGATGATATGGGACC	BamHI
pGreenII 62-SK-EIL1-R	GATAAGCTTGATATCgaattcCTGGAACCAGATCGAAACATCC	EcoRI
pGreenII 62-SK-MADS1-F	CGCTCTAGAACTAGTggatccATGGGGAGAGGAAGAGTAGAGTTG	BamHI
pGreenII 62-SK-MADS1-R	GATAAGCTTGATATCgaattcAAGCATCCAGCCAGGGATG	EcoRI
pGreenII 62-SK-MADS3-F	CGCTCTAGAACTAGTggatccATGGGGAGAGGAAGAGTGGAA	BamHI
pGreenII 62-SK-MADS3-R	GATAAGCTTGATATCgaattcTCAAAGGAGCCATCCCTGG	EcoRI
pGreenII 0800-LUC-PME1pro-F	CTATAGGGCGAATTGggtaccCGACAGCCGAGGGTCGGC	KpnI
pGreenII 0800-LUC-PME1pro-R	TGTTTTTGGCGTCTTccatggCATAGCTCCTCACATGCCTTTCT	NcoI
pGreenII 0800-LUC-PME2pro-F	CTATAGGGCGAATTGggtaccGAAGTGGAATATTGGCTTTTAAAGG	KpnI
pGreenII 0800-LUC-PME2pro-R	TGTTTTTGGCGTCTTccatggCATTGTTGATGTACTGGAGTGTGTGT	NcoI
pGreenII 0800-LUC-EXP1pro-F	CTATAGGGCGAATTGggtaccTCACATGATGGATTAATAAGCAATGT	KpnI
pGreenII 0800-LUC-EXP1pro-R	TGTTTTTGGCGTCTTccatggCATAGCTCCTCACATGCCTTTCT	NcoI
pGreenII 0800-LUC-EXP2pro-F	CTATAGGGCGAATTGggtaccATCGGATAATGAAATATATAGATGTACCTATAC	KpnI
pGreenII 0800-LUC-EXP2pro-R	TGTTTTTGGCGTCTTccatggCATAATTTCCCTCTGCTCTGTTTAGG	NcoI
pGreenII 0800-LUC-PG1pro-F	CTATAGGGCGAATTGggtaccTGTGTGTGTGTAGTTAATTTCTGCTCC	KpnI
pGreenII 0800-LUC-PG1pro-R	TGTTTTTGGCGTCTTccatggCATTGTTGAGTCGGAGAGATGAGT	NcoI
pGreenII 0800-LUC-PG2pro-F	CTATAGGGCGAATTGggtaccATATATTATGTCCTTCCCCAACTCCA	KpnI
pGreenII 0800-LUC-PG2pro-R	TGTTTTTGGCGTCTTccatggCATATATGGTTTATGAGAAATTAAATAGAGAAA	NcoI

Supplementary Table 7 Putative cis-acting element analysis for cell wall degradation related genes

gene	Cis-element	position	Core Sequence	function
CpPME1				
	ABRE	CACGTG	757(-);758(+)	abscisic acid responsiveness
	GARE	TCTGTTG	148(+)	gibberellin-responsive element
	TATC-box	TATCCCA	1044(+)	gibberellin-responsiveness
	TC-rich	GTTTTCTTAC	73(-)	stress responsiveness
	TC-rich	ATTTTCTCCA	947(+)	stress responsiveness
	TCA	GAGAAGAATA	474(-)	salicylic acid responsiveness
	LTR	CCGAAA	180(-)	low-temperature
	HSE	AAAAAATTTC	391(+);1090(-)	heat stress
CpPME2				
	ABRE	ACGTGGC	1091(-)	abscisic acid responsiveness
	CGTCA	CGTCA	830(+)	MeJA-responsiveness
	GCC-box	GCCGCC	110(-)	ethylene responsiveness
	HSE	AGAAAATTCG	82(-)	heat stress
	HSE	AAAAAATTTC	1241(-)	heat stress
	MBS	TAACTG	1314(-)	MYB binding site
	TATC-box	TATCCCA	1387(+)	gibberellin-responsiveness
	TCA	CCATCTTTTT	998(-)	salicylic acid responsiveness
	TGACG	TGACG	830(-)	MeJA-responsiveness
CpEXP1				
	ABRE	CACGTG	757(-)	abscisic acid responsiveness
	ABRE	ACGTGGC	758(+)	abscisic acid responsiveness
	GARE	TCTGTTG	148(+)	gibberellin-responsive element

	HSE	AAAAAATTTC	391(+);1090(-)	heat stress
	LTR	CCGAAA	180(-)	low-temperature responsiveness
	MRE	AACCTAA	507(-)	MYB binding site
	TATC-box	TATCCCA	1044(+)	gibberellin-responsiveness
	TA-rich	GTTTTCTTAC	73(-);947(+)	stress responsiveness
	TCA-element	GAGAAGAATA	473(-)	salicylic acid responsiveness
	W-box	TTGACC	220(-)	ethylene responsiveness
CpEXP2				
	ARE	TGGTTT	302(+)	stress responsiveness
	GARE	AAACAGA	634(+)	gibberellin-responsive element
	HSE	AAAAAATTTC	221(+);244(+)	heat stress responsiveness
	TCA-element	CAGAAAAGGA	414(-)	salicylic acid responsiveness
	TCA-element	CCATCTTTTT	505(+)	salicylic acid responsiveness
CpPG1				
	GGTCA-motif	TTTCAAA	1371(+)	MeJA-responsiveness
	GGTCA-motif	CGTCA	374(+);679(+)	MeJA-responsiveness
	HSE	AAAAAATTTC	377(-);1274(-);1197(+);1025(+);1216(-)	heat stress
	TATC-box	TATCCCA	711(+)	gibberellin-responsiveness
	TC-rich	ATTTTCTCCA	751(+)	defense and stress responsiveness
	TCA	CCATCTTTTT	1375(-)	salicylic acid responsiveness
	TGACG	TGACG	374(-);679(-)	MeJA-responsiveness
CpPG2				
	ABRE	CACGTG	620(+);623(+);622(+)	abscisic acid responsiveness
	ARE	TGGTTT	56(-);811(-);756(+)；1489(-)	anaerobic induction
	CCAAT-box	CAACGG	371(+)	MYBHv1 binding site
	GARE	AAACAGA	1449(+)	gibberellin-responsive element

HSE	AAAAAATTTC	130(+);306(+)	heat stress
MASII	AAAAGTTAGTTA	1121(+)	MYB binding site
GCC-box	GCCGCC	911(+)	ethylene responsiveness
TC-rich	ATTTTCTCCA	204(+)	defense and stress responsiveness
TCA-element	CCATCTTTTT	212(+)	salicylic acid responsiveness
TCA-element	GAGAAGAATA	1464(-);430(-)	salicylic acid responsiveness
TCCACCT	TCCACCT	38(+)	stress responsiveness

Supplementary Table 8 Significant differences between the indicators analysis of control

Indicators	Firmness	PG	PME	PL	CX	CpPG1	CpPG2	CpPME1	CpPME2	CpPME3	CpPME4	CpPL1	CpPL2	CpXYL	CpEBF1	CpEBF2	CpEIL1	CpMADS1	CpMADS3
Firmness	1																		
PG	-.897*	1																	
PME	-.907*	.820*	1																
PL	-.848*	0.802	.987**	1															
CX	-0.758	0.69	.820*	.857*	1														
CpPG1	-.752*	-.729*	0.17	-0.055	-0.21	1													
CpPG2	-0.48	-0.235	0.333	0.111	-0.04	-986*	1												
CpPME1	-0.644	0.256	.789*	-0.092	-0.22	0.785	0.786	1											
CpPME2	-0.72	0.874	0.252	0.072	0.046	0.066	0.111	0.663	1										
CpPME3	-0.037	0.601	0.075	-0.251	-0.39	0.948	0.893	0.599	-0.201	1									
CpPME4	-.970*	0.527	0.765	-0.557	-0.45	-0.568	-0.777	-0.722	-0.654	-0.277	1								
CpPL1	0.687	-0.933	-0.824	-0.905	-0.76	-0.437	-0.577	-0.549	-0.486	-0.153	.925*	1							
CpPL2	-0.202	0.584	0.475	0.626	0.28	0.86	0.929	0.813	0.347	0.663	0.896	-0.833	1						
CpXYL	0.365	0.019	-0.046	0.124	-0.31	.995**	.956*	0.807	0.903	.953*	-0.527	-0.369	0.822	1					
CpEBF1	0.898	0.424	0.351	0.51	0.112	.932*	.971**	0.871	0.326	0.769	.-827	.-719	.982*	0.91	1				
CpEBF2	.965**	0.475	0.569	0.688	0.272	0.789	0.832	.965*	0.642	0.561	-0.905	-0.746	0.921	0.784	0.934	1			
CpEIL1	.983*	0.183	0.424	0.517	0.058	0.72	0.711	.993**	0.715	0.539	.-723	0.48	0.738	0.753	0.804	0.933	1		
CpMADS1	.953*	0.285	0.555	0.629	0.202	.823*	0.633	.974*	0.814	0.405	.-774	-0.544	0.717	0.652	-0.046	-0.259	-.987*	1	
CpMADS3	-0.048	-0.851	-0.941	-0.878	-.985**	0.318	0.174	-0.098	-0.651	0.599	0.598	0.697	-0.202	0.365	-0.046	-0.259	-0.099	-0.252	1

Note: "*" represent significant correlation on 0.05, "**" represent significant correlation on 0.01

Supplementary Table 9 Significant differences between the indicators analysis of 400 nL•L^{-1} of 1-MCP for 1 h

Indicators	Firmness	PG	PME	PL	CX	CpPG1	CpPG2	CpPME1	CpPME2	CpPME3	CpPME4	CpPL1	CpPL2	CpXYL	CpEBF1	CpEBF2	CpEIL1	CpMADS1	CpMADS3
Firmness	1																		
PG	-0.307	1																	
PME	-0.583	.843*	1																
PL	-0.136	.915**	.796*	1															
CX	0.007	0.58	0.736	0.728	1														
CpPG1	-0.63	.798*	0.202	-0.187	-0.183	1													
CpPG2	-0.583	0.012	0.089	-0.289	-0.297	.991**	1												
CpPME1	-0.385	0.07	-.836*	-0.15	-0.301	.878**	.889**	1											
CpPME2	0.289	0.066	-0.347	-0.204	-0.289	0.294	0.338	0.694	1										
CpPME3	-0.752	-0.249	-0.014	-0.504	-0.552	0.603	0.643	0.344	-0.227	1									
CpPME4	-0.446	-0.501	-0.271	-0.741	-0.559	0.482	0.551	0.258	-0.145	.885**	1								
CpPL1	.798*	-.811*	-.766**	-.778*	-0.612	-0.169	-0.088	-0.191	-0.183	0.156	0.281	1							
CpPL2	0.538	0.496	0.077	0.45	0.162	-0.339	-0.339	-0.192	0.256	-0.606	-0.622	-0.052	1						
CpXYL	0.411	0.526	0.29	0.485	0.592	-0.051	-0.084	0.073	0.424	-0.634	-0.447	-0.535	0.577	1					
CpEBF1	-0.022	0.382	0.058	0.398	-0.232	-.952*	-0.064	0.153	0.266	-0.172	-0.525	-0.001	0.493	-0.179	1				
CpEBF2	0.347	0.358	-0.096	0.365	-0.182	-0.192	-0.184	0.089	0.436	-0.438	-0.637	0.064	.775*	0.143	.903**	1			
CpEIL1	-0.257	0.391	0.255	0.471	-0.052	0.036	-0.012	0.152	0.095	-0.092	-0.518	-0.094	0.216	-0.323	.918**	0.692	1		
CpMADS1	-.802*	.900*	.776*	0.487	0.351	0.086	0.009	0.196	-0.587	-.788*	0.094	-0.548	-0.334	-0.275	0.02	-0.309	0.267	1	
CpMADS3	-.951**	0.421	0.695	0.305	0.214	0.543	0.477	0.313	-0.237	0.581	0.321	-0.626	-0.547	-0.211	-0.089	-0.429	0.17	.851*	1

Note: "*" represent significant correlation on 0.05, "**" represent significant correlation on 0.01

Supplementary Table 10 Significant differences between the indicators analysis of 400 nL•L^{-1} of 1-MCP for 16 h

Indicators	Firmness	PG	PME	PL	CX	CpPG1	CpPG2	CpPME1	CpPME2	CpPME3	CpPME4	CpPL1	CpPL2	CpXYL	CpEBF1	CpEBF2	CpEIL1	CpMADS1	CpMADS3
Firmness	1																		
PG	0.145	1																	
PME	0.584	0.409	1																
PL	-0.221	0.326	0.425	1															
CX	.855*	0.252	.804*	0.201	1														
CpPG1	-.972**	-.977*	-0.606	0.271	-.860*	1													
CpPG2	-0.548	-0.068	-0.554	0.287	-0.35	0.5	1												
CpPME1	0.287	-0.298	-0.208	-.842*	-0.033	-0.417	-0.151	1											
CpPME2	0.237	-0.134	0.138	-0.478	0.228	-0.445	-0.024	.807*	1										
CpPME3	0.37	-0.1	-0.421	-0.422	-0.026	-0.221	-0.044	0.14	-0.374	1									
CpPME4	-0.436	-0.165	-0.524	0.43	-0.273	0.517	.841*	-0.487	-0.487	0.246	1								
CpPL1	0.442	-0.269	0.445	0.197	0.329	-0.294	-0.584	-0.182	-0.352	0.208	-0.218	1							
CpPL2	-.999**	-0.148	-0.597	0.216	-.872*	.978**	0.537	-0.288	-0.264	-0.34	0.438	-0.414	1						
CpXYL	-0.441	-0.003	-0.471	0.385	-0.194	0.454	.908**	-0.416	-0.271	0.076	.927**	-0.522	0.427	1					
CpEBF1	0.629	0.242	.869*	0.096	0.711	-.914*	-.831*	-0.116	0.078	-0.241	-0.695	0.479	-0.639	-0.651	1				
CpEBF2	0.565	-0.034	0.353	-0.583	0.483	-0.718	-0.416	0.748	.874*	-0.187	-0.716	-0.149	-0.59	-0.509	0.457	1			
CpEIL1	0.418	-0.496	0.49	-0.099	0.53	-0.435	-0.499	0.141	0.348	-0.303	-0.415	0.402	-0.439	-0.397	0.662	0.544	1		
CpMADS1	0.099	0.403	0.013	0.45	0.217	0.052	0.195	-.774*	-0.742	0.428	0.562	0.003	-0.095	0.541	0.016	-.751*	-0.297	1	
CpMADS3	.925**	0.247	0.693	-0.14	0.75	-.873*	-.766*	0.179	0.086	0.289	-0.61	0.619	-.914**	-0.675	.757*	0.451	0.367	0.043	1

Note: "*" represent significant correlation on 0.05, "**" represent significant correlation on 0.01
